# Remnant‐preserving anterior cruciate ligament reconstruction using hamstring autograft yields the lowest re‐rupture rates: A systematic review and meta‐analysis

**DOI:** 10.1002/jeo2.70733

**Published:** 2026-05-19

**Authors:** Ozgur Basal, James G. Jefferies, Jure Serdar, Emmanuel Papakostas, Furkan Karakas, Gazi Huri, Mahmut Nedim Doral

**Affiliations:** ^1^ Department of Orthopedics and Traumatology Medical Park Gebze Hospital Kocaeli Turkey; ^2^ Department of Orthopedics and Traumatology Maidstone & Tunbridge Wells NHS Trust London UK; ^3^ Department of Orthopedics and Traumatology University Hospital Centre Zagreb Zagreb Croatia; ^4^ Sports Orthopaedics, Aspetar Orthopaedic and Sports Medicine Hospital Doha Qatar; ^5^ Faculty of Medicine Dokuz Eylül University İzmir Turkey; ^6^ Sports Orthopaedics Aspetar Orthopaedic and Sports Medicine Hospital Doha Qatar; ^7^ Department of Orthopedics and Traumatology Magnet Hospital Ankara Turkey

**Keywords:** anterior cruciate ligament, graft failure, hamstring autograft, meta‐analysis, remnant preservation

## Abstract

**Purpose:**

Remnant‐preserving anterior cruciate ligament reconstruction (RP‐ACLR) has garnered interest due to its potential biological benefits. This study aimed to compare graft survival, complication and reoperation rates between RP‐ACLR and standard remnant‐sacrificing techniques (non‐remnant‐preserving ACLR [NP‐ACLR]), with subgroup analysis by graft type.

**Methods:**

A systematic literature search was conducted in PubMed, Embase and Cochrane Library up to June 2025. Study selection, data extraction and risk‐of‐bias assessment (using Risk of Bias In Non‐randomized Studies of Interventions [ROBINS‐I]) were performed independently by two reviewers, with discrepancies resolved by a third. Inclusion criteria comprised clinical studies (Levels I–III) reporting complications or reoperations after RP‐ACLR. Pooled rates were calculated using random‐effects meta‐analysis where appropriate.

**Results:**

Across 34 studies (*n* = 4368 patients), the RP group comprised 2759 patients and the NP group 1609 patients. Graft re‐rupture was significantly lower in the RP group (risk ratio [RR] = 0.58, 95% confidence interval [CI] 0.41–0.83), while reoperation rates did not differ significantly (RR = 1.12, 95% CI 0.56–2.22). Patient‐reported outcomes favoured RP‐ACLR, with a significant improvement in Lysholm scores. Subgroup analysis revealed the lowest re‐rupture rate with hamstring autograft and remnant preservation (2.8%). Second‐look arthroscopy demonstrated superior graft synovial coverage in RP patients (~85% vs. ~59%) and a lower incidence of cyclops lesions (pooled rate 9.4%).

**Conclusion:**

RP‐ACLR is a safe and clinically beneficial technique that demonstrates significantly lower rates of graft failure and comparable complication and reoperation rates versus standard ACLR. These findings support remnant preservation in appropriately selected patients to enhance graft survival.

**Level of Evidence:**

Level IV.

## Graphical abstract

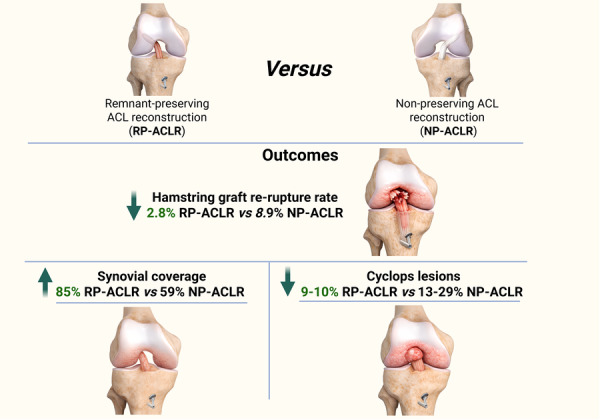

**Abbreviations**
ACL: anterior cruciate ligament
CI: confidence interval
NP: non‐preserving
RE: random‐effects
RP: remnant‐preserving
RR: risk ratio

## INTRODUCTION

Despite good to excellent outcomes in the modern era of anterior cruciate ligament reconstruction (ACLR), multifactorial risks such as graft failure, additional surgery and impaired functional or athletic return still exist [1, 2, 44]. However, continued understanding of the biological healing and mechanical processes following ACLR has led to the development and refinement of techniques aimed at improving these outcomes.

Preservation of the native tibial anterior cruciate ligament (ACL) stump during ACLR is one of the techniques hypothesized to support a number of factors that can improve healing, reduce the risk of graft rupture and ultimately lead to better outcomes for patients. The term ‘remnant preservation’ describes a broad category of techniques and pathologies that includes, but is not limited to, the location of the tear, the volume of the remaining stump and single or double bundle ACLR [23, 30, 34]. First described 25 years ago as a technique for preserving native ACL mechanoreceptors, this method is hypothesized to have a number of additional biological benefits [30, 44]. Histologically, the vascular and mechanoreceptive properties of the ACL stump remnant have been shown to play a role in restoring nerve and blood supply to the new ACL graft and facilitating fibroblast proliferation in the early healing phase [22, 32]. This additional revascularization and cell proliferation of the graft and synovial covering may allow for early graft healing and proprioception, while the increased volume of the remaining remnant may be associated with objective postoperative stability [29, 39]. Furthermore, it has been theorized that preserving the tibial stump creates a watertight barrier to the opened tibial tunnel and may reduce tunnel widening [23, 45].

Conversely, it has been suggested that remnant‐preserving (RP) techniques may lead to impingement by increasing the rate and risk of cyclops lesions in the femoral notch. However, more recent large comparative studies have shown a reversed trend towards a reduction in symptomatic cyclops in the RP‐ACLR [6, 12, 44].

In addition to reporting clinical outcomes in RP‐ACL, a recent encompassing review of systematic reviews and meta‐analyses recommended the need for robust scrutiny of the methodological quality of studies, particularly higher level studies, from which to draw meaningful conclusions to inform future practices [48].

The primary aim of this systematic review and meta‐analysis was to evaluate and compare short‐ and long‐term clinical outcomes between remnant‐preserving anterior cruciate ligament reconstruction (RP‐ACLR) and non‐remnant‐preserving ACLR (NP‐ACLR). Primary outcomes included graft re‐rupture rate, reoperation rate, cyclops lesion development and synovial coverage at second‐look arthroscopy, with subgroup analyses by graft type where appropriate. Secondary outcomes included patient‐reported outcomes (International Knee Documentation Committee [IKDC], Lysholm scores) and return to sport (RTS). It was hypothesized that RP‐ACLR would demonstrate a comparable or superior safety profile, with lower graft re‐rupture rates and without a corresponding increase in reoperation burden, compared with standard ACLR.

## METHODS

### Protocol and registration

The systematic review was previously registered in the International Prospective Registry of Systematic Reviews (PROSPERO, registration number: confidential). The study design, search and reporting were conducted in accordance with the Preferred Reporting Elements for Systematic Reviews and Meta‐Analyses (PRISMA 2020) guidelines [36].

### Eligible criteria

Eligible studies included prospective or retrospective clinical investigations (Oxford Levels I–III) evaluating RP or remnant‐resecting ACLR and reporting quantitative data on complications and/or reoperation rates. Exclusion criteria were non‐clinical studies, follow‐up <8 months, case reports, expert opinions, systematic reviews or meta‐analyses and non‐English publications.

### Information sources and search strategy

A systematic search was conducted across three electronic databases—PubMed, Embase and the Cochrane Library—from the database inception to 11 June 2025. The search strategy combined keywords related to the research topic and Medical Subject Headings (MeSH).

### Search terms

(‘Anterior Cruciate Ligament Reconstruction’ [MeSH] OR ‘ACL Reconstruction’) AND (‘Remnant Preservation’ OR ‘Stump Preservation’ OR ‘Remnant‐Preserving’) AND (‘Clinical Outcome’ OR ‘Complications’ OR ‘Reoperation’ OR ‘Revision’).

Equivalent search terms were adapted for the Embase and Cochrane databases. Only articles in English were included. No date restrictions were applied.

### Study selection process

**Figure 1:**
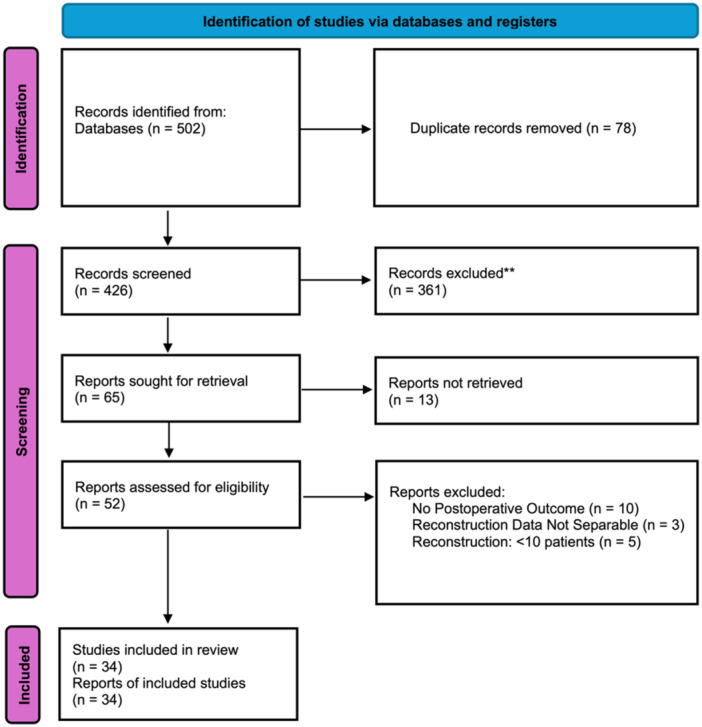
PRISMA 2020 flow diagram. Database searches of PubMed, Embase and the Cochrane Library identified 502 records. After removal of duplicates and screening, 34 studies met eligibility criteria and were included in the systematic review and meta‐analysis. PRISMA, Preferred Reporting Items for Systematic Reviews and Meta‐Analyses.

All identified articles (*n* = 502) were transferred to the online review platform (Rayyan, Qatar Computer Research Institute) [35]. Three independent reviewers performed a two‐stage selection (Figure 1):

1. Title and abstract screening: All titles and abstracts were independently screened by each reviewer according to inclusion criteria.
2. Full text evaluation: After the initial screening, 65 full‐text articles were received, and 52 met the detailed evaluation criteria.

Disputes were resolved through consensus or discussion with a senior author.

### Data collection process

Data were independently extracted by two reviewers using a standardized electronic data extraction form. Extracted variables included study characteristics (author, year, design, level of evidence), sample size and patient demographics, surgical technique (RP and NP), follow‐up period, types and frequencies of complications and reoperation rates. All data was verified by cross‐checking between reviewers.

### Data synthesis

After data extraction, studies were evaluated in terms of methodological quality and level of evidence. A total of 34 studies met all inclusion criteria and were included in the final review.

Quantitative variables were summarized as mean ± standard deviation (SD), and categorical variables as frequencies and percentages. Depending on the data distribution, comparative analyses were performed between prospective and retrospective cohorts using appropriate statistical tests.

A descriptive pooled analysis was performed to synthesize findings from heterogeneous studies. For each outcome of interest (second look rate, synovial cover and cyclops lesion incidence), data were extracted directly from the provided second look arthroscopy studies. Where possible, simple pooled percentages were calculated by summing the relevant numerators and denominators among studies reporting quantitative data. Studies reporting only qualitative findings or ‘N/A’ for a particular outcome were excluded from the pooled calculation for that outcome. The aim of the analysis was to identify significant trends and overall rates across major remnant preservation strategies (preservation and resection/standard techniques).

### Risk of bias and methodological quality assessment

**Figure 2:**
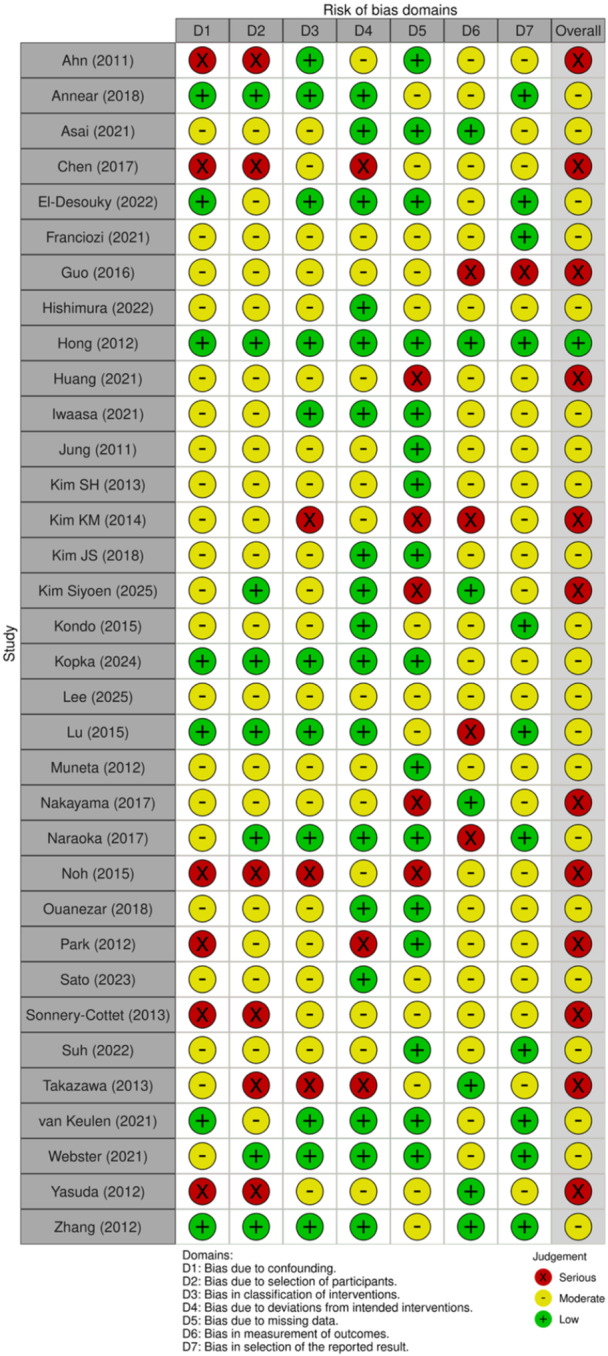
Risk of bias assessment. Summary of risk of bias ratings for all included studies using the ROBINS‐I tool (observational studies) and RoB 2 (randomized controlled trials). Traffic light plot displays domain‐level judgements (low, moderate, serious, or critical risk of bias) for each study. Domains assessed include confounding, selection of participants, classification of interventions, deviations from intended interventions, missing data, measurement of outcomes and selection of reported results. ROBINS‐I, Risk Of Bias In Non‐randomized Studies of Interventions; RoB 2, Risk of Bias 2 tool.

Risk of bias was independently assessed by two reviewers. Non‐randomized studies were assessed using the ROBINS‐I (Risk Of Bias In Non‐randomized Studies of Interventions) tool [4, 14, 15, 17, 41], which considers seven domains: (1) bias from confounding factors, (2) bias in selecting participants for the study, (3) bias in classifying interventions, (4) bias from deviations from intended interventions, (5) bias from missing data, (6) bias in measuring outcomes and (7) bias in selecting reported outcomes. Each domain was rated as low, moderate, severe or critical risk of bias according to ROBINS‐I signalling questions and guidelines. Overall risk of bias for each non‐randomized study was determined using the worst‐case principle, where the highest level of risk in any domain defined the overall assessment. When randomized controlled trials were included, risk of bias was assessed using the RoB 2 tool; for consistent presentation across study designs, domain‐level and overall assessments were summarized using a consistent colour scheme. Discrepancies between evaluators were resolved through consensus, with the inclusion of a third evaluator as needed. Results are presented in Figure 2 (a traffic light graph: green = low risk, yellow = moderate risk, red = severe risk) summarizing both field‐level and overall assessments. Studies assessed as carrying a generally severe (or critical) risk were excluded from the primary quantitative synthesis and examined only in the sensitivity analyses.

### Statistical analysis

All quantitative syntheses were performed in R (Version 4.5.2, GUI 1.82; R Foundation for Statistical Computing). Continuous outcomes (IKDC, Lysholm, KT side‐to‐side difference) were combined as MDs with 95% confidence intervals (CIs). Binary outcomes (graft re‐rupture, reoperation, cyclops lesion and complications) were summarized as risk ratios (RRs) or combined ratios, as appropriate. For comparative analyses, inverse variance‐weighted random‐effects (RE) models were applied using the constrained maximum likelihood (REML) estimator for inter‐study variance (*τ* ^2^). For small study robustness, RE CIs were calculated using the Hartung–Knapp–Sidik–Jonkman method. Combined incidence ratios were calculated using logit transformation and inversely transformed for presentation. Statistical heterogeneity was assessed using Cochran's *Q* test (*p* < 0.10 indicates significant heterogeneity) and quantified with the *I* ^2^ statistic and *τ* ^2^. *I* ^2^ values of approximately 25%, 50% and 75% were interpreted as low, moderate and high heterogeneity, respectively. Prediction ranges were calculated to estimate the expected range of effects in future comparable populations. Subgroup analyses were performed according to remnant tissue management (preserved vs. resected), graft type (hamstring autograft, bone–patellar tendon–bone autograft [BPTB], quadriceps, allograft or synthetic) and bundle configuration (single bundle vs. double bundle). Differences between subgroups were tested using mixed‐effects meta‐regression models. Meta‐regression analyses were performed to investigate potential moderators, including mean age, body mass index (BMI), time from injury to surgery, follow‐up time, graft type and remnant morphology/continuity (e.g., Crain classification). To account for the dependence between effect sizes from multi‐arm or clustered studies, robust variance estimation (CRVE) with small sample correction was applied, and results were reported as regression coefficients on a logit (for proportions) or MD scale with 95% CIs.

Sensitivity analyses were performed by (1) excluding studies with a risk of significant bias in ROBINS‐I, (2) removing outliers and impacted studies as determined by Cook distance and single exclusion diagnostics and (3) comparing co‐effects and RE models. Publication bias was assessed visually using funnel plots and statistically using the Egger regression test when at least ten studies were available. All tests were two‐sided, and unless otherwise stated, the level of statistical significance was defined as *p* < 0.05.

Artificial intelligence (AI)‐assisted writing tools were used in the preparation of this manuscript to support language editing and grammatical refinement. All scientific content, data extraction, statistical analyses, interpretation of results and conclusions remain the sole responsibility of the authors. The use of AI tools did not influence the integrity of the data or the objectivity of the reported findings.

## RESULTS

### Study characteristics

A total of 34 studies representing 4368 knees were analysed in the included cohorts; the RP group comprised 2759 patients (63.1% of the total) and the non‐remnant‐preserving (NP) group comprised 1609 patients (36.9%). Key demographic and baseline study characteristics are available in Table 1. These studies included both single‐arm and comparative trials utilizing the technique. The NP cohort was derived from comparison groups of 22 studies that directly compared both surgical procedures.

**Table 1:** Demographic and baseline characteristics of included studies.

Study	Group	*N* (knees)	Age (y)	Male	BMI	Injury to surgery (months)	Preop scores	Follow‐up (months)
Ahn et al. [1]		53	32.2	79.2% (42/53)	NR	28.2	IKDC: 59.1 ± 16.9; Lysholm: 56.2 ± 18.6	27.7
Annear et al. [3]	RP	20	28.1 ± 11.2	50.0% (10/20)	NR	1.9	NR	120
	RD	22	29.8 ± 10	59.1% (13/22)	NR	2.4	NR	120
Asai et al. [4]	P (RP)	85	25.2 ± 11.6	51.8% (44/85)	22.7 ± 2.9	NR	NR	23.8
	N (NRP)	113	22.8 ± 10.9	47.8% (54/113)	23.0 ± 3.5	NR	NR	23.8
Chen et al. [7]	Synthetic + RP	38	27.6 ± 9.3	73.7% (28/38)	NR	16.5	IKDC: 48.2 ± 9.1; Lysholm: 53.1 ± 12.4	120.8
	Hamstring autograft	73	28.6 ± 8.8	87.7% (64/73)	NR	18	IKDC: 49.5 ± 10.1; Lysholm: 50.3 ± 12.9	122.9
El‐Desouky et al. [9]	(A) RP	56	Overall 27.7 ± 7.2	92.9% (52/56)	Overall 24.4 ± 3.5	3.8	NR	Overall 25.47
	(B) NRP	53		98.1% (52/53)		3.8	NR	
Franciozi et al. [10]	F (functional)	69	31.3 ± 10.1	81%	NR	4.8	IKDC: 45.5 ± 8.0; Lysholm: 49.2 ± 8.3	31.9
	NF (nonfunctional)	75	29.6 ± 8.9	76%	NR	5.8	IKDC: 45.5 ± 7.6; Lysholm: 49.3 ± 7.8	28.6
Guo et al. [11]	Type 1 (Crain I; PCL–tibia bridge)	18	Overall: 26.7 ± 4.9	Overall: 39/51 (76.5%)	NR	Overall: 2.8	NR	Overall: Second‐look: 12.3 (10–19); Final: 53.5 (44–64)
	Type 2 (Crain II; roof–tibia bridge)	21			NR		NR	
	Type 3 (Crain III; lateral wall–tibia bridge)	4			NR		NR	
	Type 4 (Crain IV; no substantial remnant)	8			NR		NR	
Hishimura et al. [12]	A (RP)	98	29.2 ± 12.8	60.2% (59/98)	NR	6	NR	15.2
	B (NRP)	79	28.4 ± 13.4	57.0% (45/79)	NR	9.7	NR	15.2
Hong et al. [13]	Remnant‐preserving ACLR	45	34	73.3% (33/45)	NR	10.3	NR	25.8
	Standard ACLR	45	28	75.6% (34/45)	NR	9.4	NR	25.5
Huang et al. [14]	RP group (remnant preservation)	54	31.9 ± 12.7	55.6% (30/54)	24.7 ± 4.1	NR	Preınjury Tegner: 7.2 ± 1.5	37.2
	NRP group (NRP)	66	29.6 ± 12.7	42.4% (28/66)	22.7 ± 3.3	NR	Preinjury Tegner: 7.2 ± 1.3	39.6
Iwaasa et al. [15]	Preserved group	34	27.9 ± 11.4	52.9% (18/34)	23.4 ± 4.9	Median 4	Lysholm: 81.8 ± 10.4 Tegner: 5.9 ± 1.1	29
	Resected group	26	27.0 ± 11.9	34.6% (9/26)	23.8 ± 4.0	Median 4	Lysholm: 78.1 ± 12.6 Tegner: 5.6 ± 1.4	29
	Absent group	45	21.7 ± 8.8	40% (18/45)	21.8 ± 2.9	Median 3	Lysholm: 77.4 ± 13.5 Tegner: 6.0 ± 1.4	29
Jung et al. [16]	Tensioning	33	35.6 ± 13.3	81.8% (27/33)	NR	2.6	IKDC: 56.5 ± 16.4	31.5
	Preservation only	43	30.0 ± 10.5	76.7% (33/43)	NR	2.5	IKDC: 55.7 ± 16.4	32.9
Kim et al. [20]	1: Tensioning + SB	44	29.8 ± 5.4	77.3% (34/44)	NR	2.8	IKDC: 49.8 ± 16.3	45.1
	2: DB	56	30.9 ± 5.8	80.4% (45/56)	NR	2.6	IKDC: 52.6 ± 18.7	46.5
Kim et al. [18]	I ( > 50% remnant preserved)	36	32.0 ± 9.4	77.8% (28/36)	NR	~2.35	IKDC: 42.9 ± 7.2; Lysholm: 55.4 ± 8.1 Tegner: 5.1 ± 1.3	27.5
	II ( ≤ 50% remnant preserved)	30	32.0 ± 9.4	66.7% (20/30)	NR	~2.46	IKDC: 43.1 ± 5.0; Lysholm: 55.7 ± 5.4 Tegner: 5.1 ± 1.0	26.6
Kim et al. [21]	A (no remnant tissue preserved)	83	29.9 ± 10	80.7% (67/83)	NR	NR	IKDC: 58.3 ± 9.3; Lysholm: 70.1 ± 8.2 Tegner (preop): 2.5 ± 0.8	25.1
	B (remnant tissue <1/3 preserved)	38	32.2 ± 11.2	78.9% (30/38)	NR	NR	IKDC: 57.5 ± 7.4; Lysholm: 68.1 ± 11.8 Tegner (preop): 2.7 ± 0.8	25.1
	C (remnant tissue 1/3 to 2/3 preserved)	35	31.7 ± 9.5	88.6% (31/35)	NR	NR	IKDC: 57.1 ± 10.4; Lysholm: 69.5 ± 12.5 Tegner (preop): 2.5 ± 0.5	25.1
	D (remnant tissue >2/3 preserved)	29	29.1 ± 12.0	86.2% (25/29)	NR	NR	IKDC: 56.1 ± 9.4; Lysholm: 67.4 ± 11.0 Tegner (preop): 2.4 ± 0.5	25.1
Kim et al. [19]		396	26.2	83.1% (329/396)	24.3	0.95	NR	(179/396) 23.4
Kondo et al. [24]	P (RP procedure)	81	29 ± 13	54.3% (44/81)	NR	7	NR	24
	R (NRP procedure)	98	30 ± 14	55.1% (54/98)	NR	12	NR	24
Kopka et al. [25]	rACLR (remnant‐sparing ACLR)	210	34.00 ± 10.90	52.4% (110/210)	25.03	10.8	NR	24
	ACLR (SB [anatomic])	210	34.11 ± 10.81	52.4% (110/210)	25.06	14.7	NR	24
Lee et al. [26]	RC (continuity)	50	28.5 ± 6.1	80.0% (40/50)	25.5 ± 3.4	2.5	IKDC: 49.0 ± 16.7; Lysholm: 63.7 ± 19.9	35.3
	RD (discontinuity)	44	26.1 ± 8.0	81.8% (36/44)	25.9 ± 4.8	2.2	IKDC: 52.3 ± 14.5; Lysholm: 65.3 ± 20.4	36.8
Lu et al. [27]	EF group (existing footprint)	36	29.3 ± 2.4	100% (36/36)	23.78	NR	Lysholm: 65.4 ± 7.22 Tegner: 1.55 ± 0.71	34.7
	BL group (Bony landmark)	36	31.4 ± 3.1	100% (36/36)	23.79	NR	Lysholm: 66.6 ± 6.14 Tegner: 1.65 ± 0.88	39.6
Muneta et al. [29]	Well‐preserved group (≥60% remnant volume)	32	Median 25 (15–51)	46.9% (15/32)	NR	Median 2 (0.3–120)	NR	≥24
	Moderately preserved group (35%–55% remnant volume)	26	Median 22 (14–41)	38.5% (10/26)	NR	Median 3 (1–24)	NR	≥24
	Less‐preserved group (≤30% remnant volume)	30	Median 19 (14–48)	43.3% (13/30)	NR	Median 14 (2–240)	NR	≥24
Nakayama et al. [31]	Preservation group	50	26.6 ± 9.7	56.0% (28/50)	NR	NR	NR	Min 12
	NRP	75	26.4 ± 11.7	53.3% (40/75)	NR	NR	NR	Min 12
Naraoka et al. [32]	RP group (remnant‐preserved group)	77	25.1 ± 11.7	28.6% (22/77)	NR	NR	NR	Min 24
	NR group (nonremnant group)	74	20.1 ± 9.0	33.8% (25/74)	NR	NR	NR	Min 24
Noh et al. [33]		43	25 ± 7	69.8% (30/43)	NR	0.9	Lysholm: 54 ± 11	25.7
Ouanezar et al. [34]		128	35	64% (82/128)	NR	16.7	IKDC: 54.1 ± 13.6	31.7
Park et al. [37]	Aug group (RP augmentation)	55	30.4	81.8% (45/55)	NR	7	IKDC: 47.1 ± 16.3; Lysholm: 48.6 ± 9.7	34.1
	DB group (DB)	45	31.7	88.9% (40/45)	NR	11	IKDC: 48.8 ± 17.4; Lysholm: 48.6 ± 8.7	30.8
Sato et al. [38]	Anatomical attachment (AA)	34	36.5 ± 15.1	55.9% (19/34)	22.7 ± 2.5	~1.7	KOOS: 92.1 ± 6.4	24
	Non‐anatomic	33	30.0 ± 14.6	48.5% (16/33)	23.4 ± 4.1	~1.8	KOOS: 87.0 ± 9.7	24
	No remnant	22	22.1 ± 11.0	31.8% (7/22)	23.3 ± 4.3	~2.5	KOOS: 86.4 ± 12.3	24
Sonnery‐Cottet et al. [40]		39	30 ± 10.2	56.4% (22/39)	NR	5.7 ± 6.8	IKDC: 43.5 ± 16.6; Lysholm: 60.8 (17–89)	24.2 ± 4.2
Suh et al. [41]	R (preservation)	42	33	88.1% (37/42)	25.4	0.6	NR	24
	C (control)	22	39	81.8% (18/22)	24.6	0.4	NR	24
Takazawa et al. [43]	One (remnant preserved)	85	24.3 ± 8.4	NR	NR	7.3 ± 16.3	NR	33.3 ± 10
	Two (remnant not preserved)	98	26.1 ± 8.3	NR	NR	16.0 ± 30.3	NR	31.0 ± 9.8
van Keulen et al. [17]	SRTP	67	23 ± 7	58% (39/67)	23 ± 3	3.5 mo (*n* = 65)	IKDC: 64 ± 15; KOOS: (*n* = 54) QoL 49 ± 18; Sports 48 ± 28; Symptoms 70 ± 21; ADL 90 ± 13; Pain 79 ± 16	24
	SR	67	23 ± 7	58% (39/67)	24 ± 3	3.2 mo (*n* = 60)	IKDC: 58 ± 13 (*n* = 50); KOOS: (n = 54) QoL 47 ± 25; Sports 41 ± 28; Symptoms 67 ± 23; ADL 82 ± 16; Pain 72 ± 17	24
Webster et al. [44]	No stump	228	26.3 ± 10	66.7% (152/228)	NR	2.2 ± 1.5	NR	24
	<50% stump	342	26.0 ± 9	59.9% (205/342)	NR	2.3 ± 1.4	NR	24
	>50% stump	88	26.0 ± 9	54.5% (48/88)	NR	2.2 ± 1.2	NR	24
Yasuda et al. [46]		44	29	61.4% (27/44)	NR	4	NR	16.6
Zhang et al. [47]	RP	27	23.5 ± 4.2	70.4% (19/27)	NR	12.7 ± 11.6	Lysholm 60.3 ± 5.3 (51–69)	24.4 ± 0.8
	RD	24	25.3 ± 6.1	87.5% (21/24)	NR	10.2 ± 9.0	Lysholm 58.7 ± 6.5 (48–71)	25.2 ± 1.1

*Note*: Study‐level and group‐level data reporting sample size (knees), age (years), sex (% male), BMI (kg/m^2^), time from injury to surgery (months), preoperative patient‐reported outcome scores and follow‐up duration (months). Data are presented as mean ± SD or median (range/IQR) as reported. Injury‐to‐surgery intervals are expressed in months (weekly values converted). Preoperative scores include only outcomes explicitly reported in each study. Group labels follow original study terminology.

Abbreviations: ACLR, anterior cruciate ligament reconstruction; ADL, Activities of Daily Living; BMI, body mass index; DB, double‐bundle; IKDC, International Knee Documentation Committee; IQR, interquartile range; KOOS, Knee Injury and Osteoarthritis Outcome Score; NP‐ACLR, non‐remnant‐preserving ACLR; NR, not reported; NRP, non‐remnant preservation; PCL, posterior cruciate ligament; QoL, Quality of Life; RD, remnant debridement; RP, remnant preservation; SB, single‐bundle; SD, standard deviation; SRTP, standard reconstruction with tissue preservation.

This distribution demonstrates that remnant‐sparing techniques have been extensively studied and applied in clinical settings in the reviewed literature. Therefore, out of all 34 studies, 27 (79.4%) used a comparative design, while the remaining 7 were single‐arm cohort studies reporting outcomes for a specific remnant‐sparing technique. A total of 4368 knees were analysed in the included cohorts;

The weighted mean patient age was 28.4 years (interstudy range approximately 20–39 years) and the overall male proportion was 65.7%. The weighted mean time from injury to surgery was 6.8 months, but significant within‐study variability was observed, ranging from acute reconstructions performed within weeks to delayed procedures lasting more than a year. The weighted mean follow‐up time was 42.3 months, with several long‐term series exceeding 10 years.

BMI had a mean of 24.2 kg/m^2^. BMI and rupture risk were not found to be related following meta‐regression of seven studies (*β* = 0.0068; 95% CI− 0.1776 to 0.1912; *p* = 0.94). The study cohort had a pooled mean BMI of 24.2 kg/m^2^. The relationship between remnant preservation and re‐rupture was unaffected by BMI within this small range (≈22–25 kg/m^2^). However, limited BMI variability and study number likely precluded detection of effects in overweight or obese populations.

Heterogeneously reported baseline functional status showed moderate preoperative impairment. Based on data reporting this outcome from the studies, the weighted mean subjective IKDC score was 52.5, and the weighted mean Lysholm score was 61.2. The Tegner activity level showed a weighted mean of 4.6, indicating participation in recreational or moderately competitive sports prior to surgery. KOOS scores were reported inconsistently and were often given as subscale values, which prevented a reliable overall total score calculation.

### Risk of bias assessment

The included studies consisted of 6 randomized controlled trials (RCTs), 6 prospective cohort or prospective comparative studies, 17 retrospective cohort or retrospective comparative studies and 5 case series. Overall risk of bias was assessed using the ROBINS‐I tool and is summarized in Figure 2.

The quality of evidence for included studies was deemed at moderate or serious risk of bias overall. The most frequent sources of potential bias were confounding (*n* = 26), selection of participants (*n* = 26) and missing data (*n* = 18). Randomized and well‐controlled prospective studies generally demonstrated more favourable risk‐of‐bias profiles, whereas retrospective cohorts and case series showed higher risk, with several studies exhibiting serious concerns related to baseline imbalance, incomplete follow‐up and deviations from intended interventions.

Based on the overall ROBINS‐I assessment using the worst‐case scenario principle, studies judged to have a serious or critical risk of bias were excluded from the primary meta‐analysis and considered only in sensitivity analyses. Consequently, the main quantitative synthesis was driven by studies with a low to moderate risk of bias, strengthening the internal validity of the pooled estimates.

The traffic‐light visualization displays domain‐level and overall ROBINS‐I judgments, with green indicating low risk of bias, yellow indicating moderate risk and red indicating serious risk. Overall risk of bias for each study was assigned according to the worst‐domain approach.

### Graft characteristics

Twenty‐two studies (comprising 2044 patients) used hamstring tendon autografts, which was the predominant graft choice used in RP‐ACL. BPTB autograft or allograft was used in four studies (329 patients), quadriceps tendon autograft in one study (53 patients) and Achilles tendon allograft in three studies (475 patients). A summary of graft choice and surgical technique including remnant preservation of included studies is presented in Table 2.

**Table 2:** Surgical characteristics and remnant management strategies.

Study	Reconstruction	Graft	Remnant strategy
Ahn et al. [1]	SB ACLR (four‐strand)	Hamstring autograft; four‐strand	Preserve tibial stump; suture and femoral tensioning during fixation
Annear et al. [3]	SB ACLR	ST + G autograft; double‐loop	Minimal: debride unstable fibres only; spare stable remnant + notch synovium/fat pad/lig. mucosum. Standard: remove all remnant + notch soft tissue
Asai et al. [4]	Anatomic SB ACLR	Hamstring autograft	Crain II–III: preserve femoral‐side remnant; drill femoral tunnel behind remnant (minimal peeling). Crain I/IV: excise proximal remnant
Chen et al. [7]	SB (one femoral + one tibial tunnel)	(1) LARS AC120 (synthetic) (2) ST + G autograft; four‐strand	(1) Preserve stump; drill tibial tunnel through stump (K‐wire through stump). (2) NR
El‐Desouky et al. [9]	NR	Hamstring autograft	Group A: preserve tibial stump; suture stump to graft. Group B: debride femoral + tibial stumps (resect)
Franciozi et al. [10]	Anatomic SB (transportal)	ST + G autograft; quintuple (tripled ST + doubled G)	Functional fibres (Type III/IV): pass graft alongside remnant; preserve functional fibres/femoral attachment; minor tunnel adjustment. Nonfunctional (Type I/II): sleeve technique; keep remnant as sleeve and create central channel for graft
Guo et al. [11]	SB	BPTB allograft	Crain I–III: remnant‐preserving. Crain IV: no remnant (N/A)
Hishimura et al. [12]	Anatomic DB (AM + PL)	Doubled ST autograft + Leeds‐Keio tape (hybrid)	Preserved: create tunnels through/around remnant without detaching proximal attachment. Resected: remove remnant before tibial tunnel creation
Hong et al. [13]	SB	Allograft (tibialis anterior or hamstring), four‐strand	Preservation arm: preserve + tension remnant; suture remnant to graft (two stitches) as a unit. Control: standard ACLR (no preservation/tensioning)
Huang et al. [14]	SB	ST autograft; 4–5 strands	RP: preserve if femoral attachment intact, >50% volume, synovial coverage with blood supply. NRP: debride/clean if criteria not met
Iwaasa et al. [15]	Anatomic DB (AM + PL)	Hamstring autograft (ST; ± G)	Preserved: attempt RP tunnel creation; continuity retained >50%. Resected: continuity lost during tunnel creation ‐> remove remaining remnant. Absent: Crain IV (no continuity) ‐> remove remaining remnant
Jung et al. [16]	SB (AM bundle)	Hamstring autograft; four‐strand	Tensioning group: preserve remnant + pullout suture tensioning tied over lateral femur. Preservation‐only: preserve remnant without tensioning
Kim et al. [20]	(1) SB AM + remnant tensioning (tensioned bridging) (2) DB ACLR	(1) Hamstring (ST for AM, G for PL) (2) three‐strand ST (AM) + three‐strand G (PL)	(1) Preserve remnant; tension with PDS sutures via two small femoral tunnels, tied over lateral cortex (toward PL). (2) NR
Kim et al. [18]	SB (transtibial; femoral tunnel between AM/PL attachments)	Hamstring autograft	Remnant‐preserving: pass graft through remnant; position remnant lateral to graft to guide PL insertion. Groups: >50% versus <50% remnant preservation possible
Kim et al. [21]	SB	BPTB autograft	Classify remnant after debridement: none/<1/3/1/3‐2/3/ > 2/3 preserved
Kim et al. [19]	NR	Achilles tendon allograft (bone plug tibial side)	Preserve remnant tissue with sutures; tie remnant to graft
Kondo et al. [24]	Anatomic DB (AM + PL)	ST autograft + Leeds‐Keio tape (hybrid)	Preserve remnant: create fibre‐aligned slits and drill tunnels while maintaining attachments. Type IV: resect before tibial tunnels
Kopka et al. [25]	SB	Hamstring autograft or fresh‐frozen soft‐tissue allograft	Remnant‐sparing if robust tibial stump >50% and/or fibres still attached at femoral origin. Otherwise standard SB ACLR
Lee et al. [26]	NR	Hamstring autograft; four‐strand double‐loop (5–6 strands if <7 mm)	Remnant repair/tensioning with three PDS stitches tied over suspensory device after fixation
Lu et al. [27]	DB (AM + PL), four‐tunnel	Hamstring autograft (ST + G; contralateral G if needed)	Preservation arm: preserve remnant and use fibre direction to individualize footprint centres for tunnel placement. Control: clean notch; place tunnels using bony landmarks
Muneta et al. [29]	Anatomic DB (AM + PL; trans‐tibial)	ST autograft; four‐strand	Preserve tibial remnant as much as possible; peel only remnant tissue at normal femoral attachment
Nakayama et al. [31]	Anatomic DB (AM + PL)	Hamstring autograft (ST); two double‐strand grafts	Preservation arm: keep full‐volume remnant; tension with traction sutures through femoral tunnel tied over EndoButton; position remnant between AM/PL grafts. Control: standard debridement
Naraoka et al. [32]	Anatomic DB	ST autograft	Keep tibial remnant in situ; remove femoral‐side remnant; pass grafts through any remaining remnant tissue
Noh et al. [33]	SB	Achilles allograft; two‐strand looped	Preserve remnant and re‐tension/cover graft using tethered PDS sutures
Ouanezar et al. [34]	SB anatomic (SAMBBA)	ST autograft with tibial attachment preserved; tripled/quadrupled	Minimal notch debridement; pass graft through ACL remnant
Park et al. [37]	Selective augmentation (AM or PL)/DB	Hamstring autograft (augmentation)/mixed (DB arm)	Augmentation: preserve remnant bundle and augment deficient bundle. DB arm: NR
Sato et al. [38]	Anatomic DB (AM + PL)	ST autograft (split + doubled)	Behind‐remnant: leave femoral + tibial remnant intact; place grafts through/covered by remnant. Crain IV: no femoral remnant (N/A)
Sonnery‐Cottet et al. [40]	Selective PL reconstruction/augmentation (AM remnant preserved)	ST autograft (tibial insertion preserved); doubled/tripled	Preserve AM bundle remnant; no notchplasty
Suh et al. [41]	Group R (RP) versus Group C (control)	Allo‐Achilles tendon	RP: protect remnant during reaming with sutures; after fixation, tension/suture remnant directly to graft. Control: no remnant preservation
Takazawa et al. [43]	SB	ST autograft + Telos artificial ligament (composite); five‐strand	Preserved: met post‐insertion definition (vascular synovial coverage, >75% graft coverage from tibial attachment, femur‐tibia bridge). Not preserved: attempted but definition not met
van Keulen et al. [17]	SB all‐inside	ST autograft; quadrupled	SRTP: keep continuous but insufficient remnant ACL (AM); debride only as needed for socket prep. SR: no tissue could be spared; debride remnants
Webster et al. [44]	SB	ST + G autograft (doubled; ST may be tripled)	Classify tibial stump after passage/final debridement: no stump/<50% coverage/>50% coverage
Yasuda et al. [46]	Anatomic DB (AM + PL)	ST autograft + Leeds‐Keio tape (hybrid)	Remnant preserved throughout (no conversion to resecting technique)
Zhang et al. [47]	SB (RP vs. resection)	Hamstring autograft; quadruple‐loop	RP: preserve as completely as possible; partial debridement only if flipped anteriorly/sclerotic (cyclops prevention). Control: tibial remnant completely resected

*Note*: Summary of surgical techniques, graft types, bundle configurations, fixation methods and remnant management approaches across included studies. Remnant classification systems (e.g., Crain grade, remnant continuity, remnant volume) are reported where applicable. Where studies included multiple surgical groups, each group is described separately.

Abbreviations: ACLR, anterior cruciate ligament reconstruction; AM, anteromedial; BPTB, bone–patellar tendon–bone autograft; DB, double‐bundle; G, gracilis; HS, hamstring autograft; LARS, ligament augmentation and reconstruction system; N/A, not applicable; NP‐ACLR, non‐remnant‐preserving ACLR; NR, not reported; QB, quadriceps bone autograft; PDS, polydioxanone; PL, posterolateral; RP, remnant‐preserving; SB, single‐bundle; SRTP, standard reconstruction with tissue preservation; ST, semitendinosus.

RP techniques ranged from limited debridement and tibial stump preservation to remnant stretching, sleeve techniques and graft passage through preserved tissue. Reconstructions were performed using single‐bundle, double‐bundle or selective bundle techniques. When available, remnant handling was guided by morphology or continuity classifications (e.g., Crain types) or study‐specific thresholds (e.g., stump volume or coverage).

Surgical techniques, graft configurations and remnant‐preservation strategies across included studies are summarized in Table 2.

### Meta analyses

#### Remnant preservation versus non‐preservation: IKDC and Lysholm outcomes

**Figure 3:**
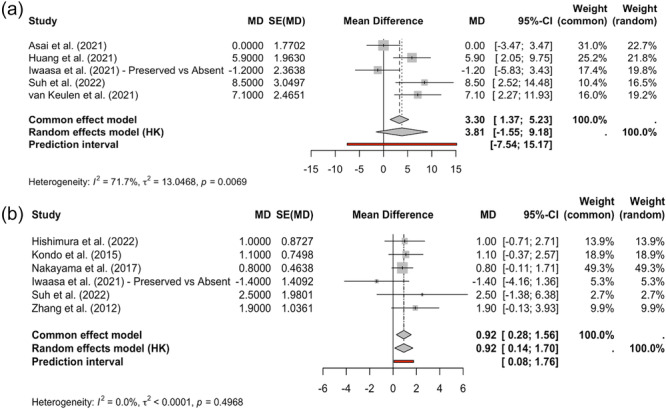
Effect of remnant preservation on IKDC and Lysholm scores after ACLR. Forest plots comparing remnant‐preserving versus non‐remnant‐preserving ACLR for postoperative (a) IKDC scores (upper panel) and (b) Lysholm scores (lower panel), expressed as pooled MDs with 95% CIs. Diamonds show combined effects under the fixed‐effects model and the Hartung–Knapp random‐effects model; horizontal bars indicate 95% prediction intervals. Positive MD values support remnant preservation. Inter‐study heterogeneity is reported using *I* ^2^ and *τ* ^2^ statistics. ACL, anterior cruciate ligament; CI, confidence interval; *I* ^2^, heterogeneity statistic; IKDC, International Knee Documentation Committee; MD, mean difference; *τ* ^2^, between‐study variance.

Five studies reported relevant comparative data for final IKDC scores between RP and NP or remnant‐free groups (Asai et al., Huang et al., Iwaasa et al., Suh et al. and van Keulen et al.) [4, 14, 15, 17, 41]. In a fixed‐effects model, RP was associated with a higher IKDC score (MD, 3.30 points; 95% CI, 1.37–5.23). However, heterogeneity was significant (*I* ^2^ = 71.7%, *τ* ^2^ = 13.05; *p* = 0.0069), suggesting significant differences between studies. Accordingly, under a Hartung–Knapp RE model, the combined effect remained directionally favourable but not statistically significant (MD, 3.81; 95% CI, −1.55 to 9.18). The wide prediction range (−7.54 to 15.17) indicates that the expected effect can vary significantly across clinical settings and surgical techniques, ranging from no benefit (or worse outcomes) to clinically significant improvement (Figure 3a).

Six studies reported suitable Lysholm scores for quantitative synthesis [12, 15, 24, 30, 41, 47]. Unlike the IKDC analysis, no heterogeneity was observed (*I* ^2^ = 0%, *τ* ^2^ < 0.0001, *p* = 0.50). Both fixed‐effects and Hartung–Knapp RE models showed a small but statistically significant improvement in Lysholm scores in the RP group (MD = 0.92 points; 95% CI, 0.28–1.56 and 0.14–1.70, respectively) (Figure 3b). The prediction range remained entirely on the side supporting remnant preservation (0.08–1.76), suggesting that the functional advantage is consistent in future comparable populations. Collectively, these findings suggest that remnant preservation is associated with a modest and consistent improvement in Lysholm functional outcomes, whereas its effect on IKDC scores is more heterogeneous and dependent on study‐level factors, showing a benefit trend that does not maintain robustness under RE modelling.

#### Remnant preservation versus non‐preservation: Graft re‐rupture and reoperation rates

**Figure 4:**
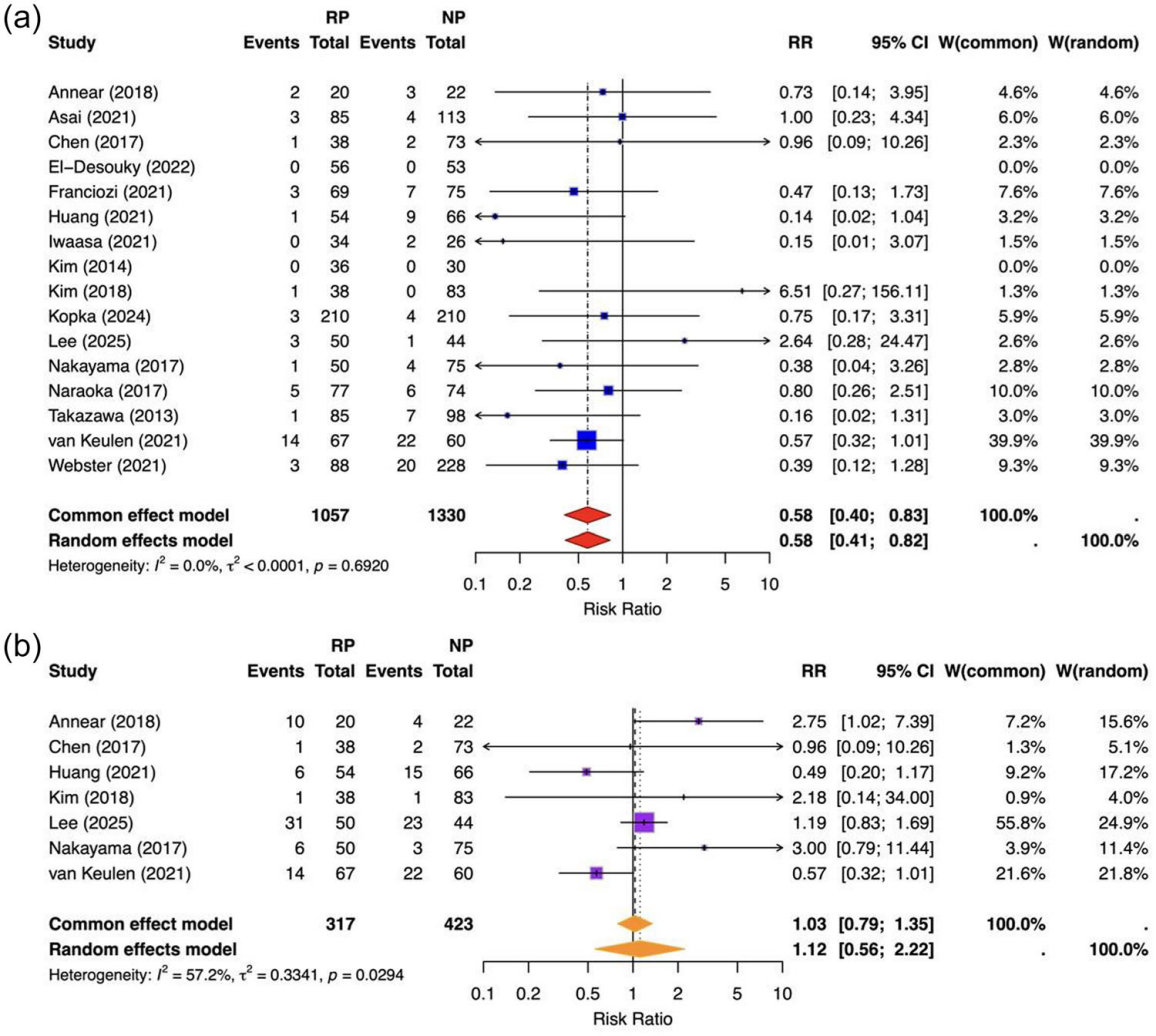
Forest plots for primary safety outcomes. Comparisons of remnant‐preserving versus non‐remnant‐preserving ACLR for (a) graft re‐rupture (upper panel) and (b) reoperation rates (lower panel). Squares represent study‐level RRs with 95% CIs; diamonds indicate the pooled effect estimate (random‐effects model). Values < 1 favour remnant preservation. ACL, anterior cruciate ligament; CI, confidence interval; RR, risk ratio.

In a combined analysis of comparative studies, remnant‐sparing ACLR was associated with a significantly lower risk of graft re‐rupture compared to non‐sparing techniques (RE RR = 0.58, 95% CI 0.41–0.83; *I* ^2^ = 0%), representing a 42% relative risk reduction with minimal inter‐study heterogeneity. In contrast, no significant difference was observed between groups in terms of overall reoperation rates (random effects RR = 1.12, 95% CI 0.56–2.22; *I* ^2^ = 57.2%), indicating significant heterogeneity reflecting variability in surgical indications and follow‐up time between studies (Figure 4). These findings suggest that remnant preservation may improve graft survival without a corresponding increase in secondary surgical procedures.

**Figure 5:**
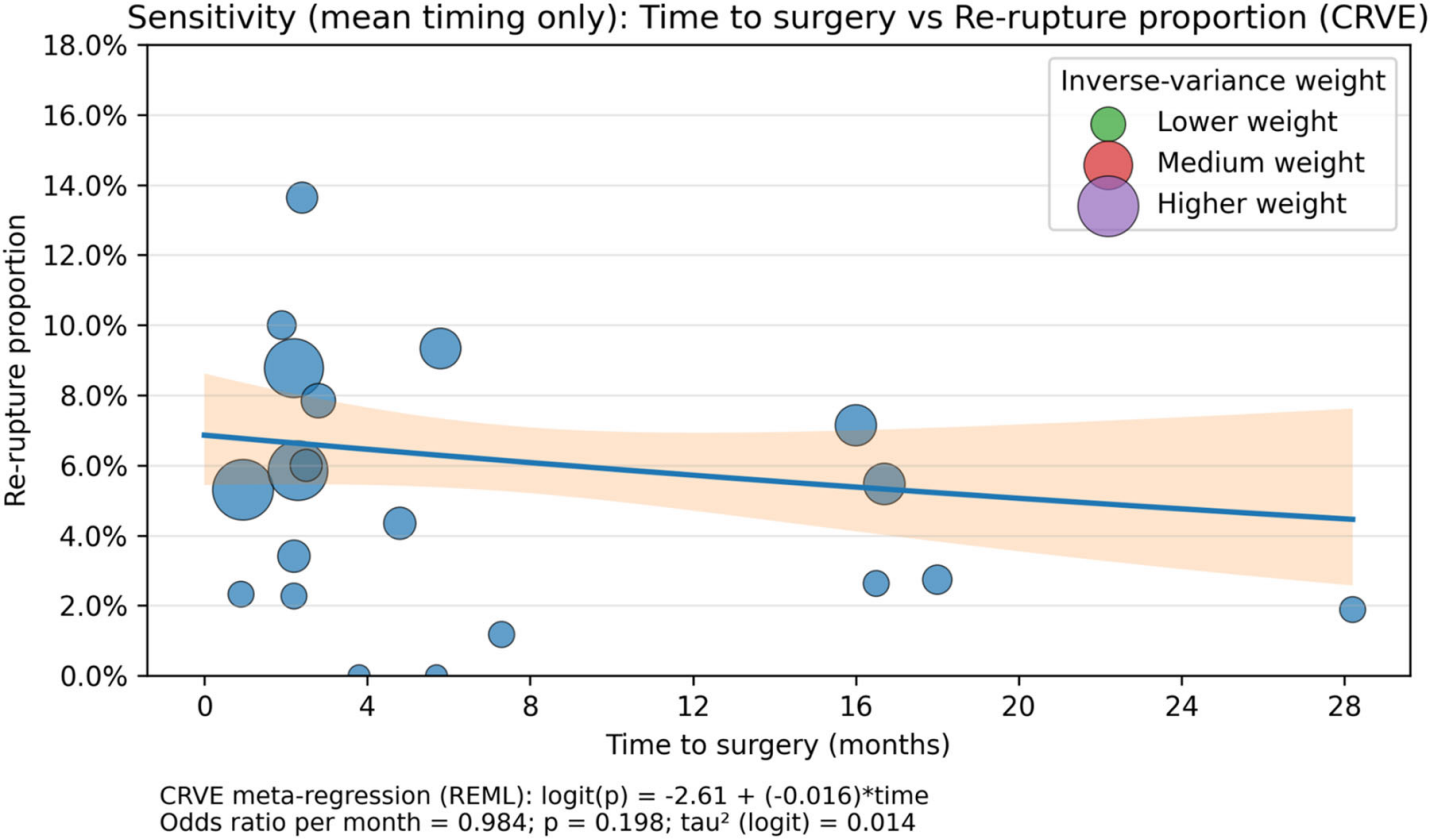
Meta‐regression: time from injury to surgery and re‐rupture rate. Weighted bubble plot showing the association between mean time from injury to ACLR (months) and the re‐rupture proportion across included studies. Bubble size is proportional to inverse‐variance weight. The regression line was estimated using cluster‐robust variance estimation (CRVE) with REML: logit(*p*) = −2.61 + (−0.016) × time; odds ratio per month = 0.984; *p* = 0.198; *τ* ^2^ (logit) = 0.014. The shaded band represents the 95% CI around the regression line. ACL, anterior cruciate ligament; CI, confidence interval; CRVE, cluster‐robust variance estimation; REML, restricted maximum likelihood; *τ* ^2^, between‐study variance.

In the CRVE‐adjusted meta‐regression restricted to studies reporting mean time from injury to surgery, surgical delay was not significantly associated with re‐rupture risk. The estimated logit‐scale slope was −0.016 per month (equivalent to an odds ratio of 0.984 per month), suggesting only a minimal, statistically non‐significant reduction in re‐rupture odds with longer delay (*p* = 0.198). Remnant between‐study heterogeneity after accounting for the moderator was low (*τ* ^2^ = 0.014). Consistent with this, the weighted bubble plot showed only a slight inverse pattern, without evidence of a meaningful dose–response relationship (Figure 5).

#### Double versus single bundle reconstruction and its effect on side‐to‐side difference

Meta‐regression examining bundle configuration across the included studies demonstrated that double‐bundle (DB) ACLR was associated with a slight reduction in postoperative anteroposterior laxity compared to single‐bundle (SB) techniques, corresponding to a reduction of approximately 0.4–0.6 mm in the KT‐1000/2000 side‐to‐side difference. While this absolute difference was small and below the clinically significant minimum threshold (≈1 mm) for most patients, the direction of effect was generally consistent and consistent with the biomechanical rationale that anatomical DB reconstruction may better regenerate native ACL function and knee stability. Clinically, the potential gain in objective stability should be interpreted in conjunction with the greater technical requirements of DB reconstruction and the lack of a consistent, clinically significant superiority in patient‐reported outcomes across studies.

#### ACL re‐rupture rates regarding graft type and remnant preservation status

A comprehensive meta‐analysis of 24 studies encompassing 2327 ACLRs revealed several key findings. First, using random effects modelling, the overall combined re‐rupture rate was 5.1% (95% CI 4.2–6.1) across all graft types. When graft types were examined the graft types separately, the re‐rupture rate was found to be 4.92% in hamstring autografts (95% CI 3.75%–6.22%, 20 studies, *n* = 1799), 4.57% in Achilles tendon allografts (95% CI 2.48%–7.18%, 2 studies, *n* = 439), 8.11% in BPTB allografts (95% CI 1.93%–17.5%, 1 study, *n* = 51) and 3.17% in synthetic ligament augmentation and reconstruction system (LARS) grafts (95% CI 0%–11.6%, 1 study, *n* = 38). The Kruskal–Wallis test revealed no statistically significant difference between these graft types (*H* = 1.836, *p* = 0.607). In a randomized effects meta‐analysis of mass graft re‐rupture rates, significant differences were observed according to graft type and remnant management strategy. Hamstring autograft with remnant preservation showed the lowest mass re‐rupture rate at 2.8% (95% CI, 1.8–4.1), with low interstudy heterogeneity (*I* ^2 ^= 12%). In contrast, hamstring autograft without remnant preservation showed a significantly higher failure rate of 8.9% (95% CI, 6.7–11.6; *I* ^2^ = 25%). Among allografts, Achilles tendon allograft using remnant preservation technique showed a moderate re‐rupture rate of 5.0% (95% CI, 3.2–7.4; *I* ^2 ^= 18%), while bone–patellar tendon–bone allograft showed a higher and more heterogeneous failure rate of 7.8% (95% CI, 2.2–18.9; *I* ^2^ = 45%). Synthetic ligament reconstruction (LARS) was associated with a re‐rupture rate of 2.6% (95% CI, 0.1–13.5); however, the wide CI and significant heterogeneity (*I* ^2^ = 68%) reflect the small sample size and limited number of studies available (Table 3). Overall, when all grafts and techniques were combined, the summary re‐rupture rate was 4.2% (95% CI, 3.1–5.6), indicating moderate heterogeneity (*I* ^2^ = 42.5%, *p* = 0.03).

**Table 3:** Pooled re‐rupture rates after ACLR by graft type and remnant preservation strategy.

Graft type	Total knees (*n*)	Total re‐ruptures	Pooled rate (%, RE)	Average follow‐up
Hamstring autograft—remnant preserved	~850	~24	≈2.8% (95% CI ≈ 1.8–4.1)	24–41 months
Hamstring autograft—non‐preserved/poor remnant	~700	~63	≈8.9% (95% CI ≈ 6.7–11.6)	24–39 months
BPTB allograft	51	4	≈7.8% (95% CI ≈ 2.2–18.9)	~31 months
Achilles tendon allograft (RP)	439	22	≈5.0% (95% CI ≈ 3.2–7.4)	28–60 months
Synthetic (LARS)	38	1	≈2.6% (95% CI ≈ 0.1–13.5)	~41 months

*Note*: Subgroup meta‐analysis of graft re‐rupture rates stratified by graft type (hamstring autograft, BPTB, allograft and other) and remnant preservation status. Pooled rates, 95% CIs, *I* ^2^ statistics and average follow‐up are presented for each subgroup. Random‐effects model (Hartung–Knapp adjustment).

Abbreviations: ACL, anterior cruciate ligament; BPTB, bone–patellar tendon–bone; CI, confidence interval; *I* ^2^, heterogeneity statistic; LARS, ligament augmentation and reconstruction system; RE, random‐effects; RP, remnant‐preserving.

#### Second‐look arthroscopic findings by remnant preservation techniques

A total of 12 studies involving 1129 patients undergoing ACLR were included in the analysis of second‐look arthroscopic outcomes. Of these, 797 patients (70.5%) underwent a scheduled second‐look arthroscopy at an average time interval ranging from 12 to 32.7 months postoperatively. Arthroscopic evaluation revealed favourable graft synovialization, consistent with ‘good’ or ‘excellent’ coverage in 53.8% to 92.9% of cases. As shown in comparative studies such as Nakayama et al., remnant‐sparing techniques were associated with significantly superior synovial coverage compared to non‐sparing approaches, where 92.9% coverage was achieved in the sparing group and 59.1% in the non‐sparing group [30]. The incidence of cyclops lesions ranged from approximately 10% to 22% across the studies. Lower cyclops rates were observed in cohorts with significant synovial covering and remnant integrity, as reported by Hong et al. (10.9%), Sato et al. (10.1%) and Lee et al. (14.8%) [13, 26, 38]. In contrast, lower synovial covering and higher cyclops formation rates were seen in cohorts with remnant resection or poor integrity. Overall, second‐look arthroscopic findings suggest that remnant‐sparing ACLR is associated with superior synovial covering of the graft, improved graft‐remnant integration and a trend toward lower cyclops lesion formation compared to non‐sparing techniques (Table 4).

**Table 4:** Quantitative second‐look arthroscopic outcomes by remnant preservation strategy.

Study	Group	Sample size	Second look arthroscopy (*n*, %)	Timing (months)	Graft type	Synovial coverage % (good/fair)
Ahn et al. [1]	Remnant preservation	53	33 (62.3%)	≥12	Four‐strand hamstring autograft	30/33 (90.9%)
Hong et al. [13]	Study group (remnant preservation)	39	28 (71.8%)	≥24	Four‐strand allograft (tibialis anterior or hamstring tendons)	Good (>75%): 60.7% (17/28); Fair (50%–75%): 10.7% (3/28)
	Control group (standard ACLR)	41	27 (65.9%)	≥24	Four‐strand allograft (tibialis anterior or hamstring tendons)	Good (>75%): 59.3% (16/27); Fair (50%–75%): 11.1% (3/27)
Kim et al. [20]	Group 1: SB + remnant tensioning/preservation	44	15 (34.1%)	Not specified	Hamstring (quadrupled graft)	Good (excellent): 80% (12/15); Fair: 20% (3/15)
	Group 2: Double‐bundle ACLR	56	11 (19.6%)	Not specified	ST (three‐strand) for AM + gracilis (three‐strand) for PL	Good (excellent): 18% (2/11); Fair: 54% (6/11)
Noh et al. [33]	Single cohort (remnant‐preserving + re‐tensioning)	43	43 (100%)	≥12	Two‐strand Achilles allograft	Good (>75%): 39/43 (90.7%) Fair (50%–75%): 3/43 (7.0%)
Guo et al. [11]	Crane Type 1 (PCL‐tibia)	18	18 (100%)	12.3	BPTB allograft	Good (>75%): 50.0% (9/18); Fair (50%–75%): 16.7% (3/18)
	Crain Type 2 (roof‐tibia)	21	21 (100%)	12.3	BPTB allograft	Good: 38.1% (8/21); Fair: 42.9% (9/21)
	Crain Type 3 (lateral wall‐tibia)	4	4 (100%)	12.3	BPTB allograft	Good: 0% (0/4); Fair: 25.0% (1/4)
	Crain Type 4 (no substantial remnant)	8	8 (100%)	12.3	BPTB allograft	Good: 25.0% (2/8); Fair: 0% (0/8)
Kim et al. [18]	Group 1 (remnant preserved >50%)	36	36/36 (100%)	~27	Hamstring autograft	Good: 29/36 (80.6%) Fair (partial/‘half’): 7/36 (19.4%)
	Group 2 (remnant preserved ≤50%)	30	30/30 (100%)	~27	Hamstring autograft	Good: 7/30 (23.3%) Fair (partial/‘half’): 20/30 (66.7%)
Hishimura et al. [12]	Group A (Crain Type I–III; remnant‐preserving DB‐ACLR)	98	98/98 (100%)	15.2 ± 8.4	Hamstring autograft	Good (Grade A = completely covered): 91/98 (92.9%) Fair (Grade B = partially covered): 6/98 (6.1%)
	Group B (Crain Type IV; remnant resected DB‐ACLR)	79	79/79 (100%)	15.2 ± 8.4	Autogenous semitendinosus tendon (cut in half, doubled; AM/PL bundles) + polyester tape; EndoButton fixation	Good (Grade A): 63/79 (79.7%) Fair (Grade B): 14/79 (17.7%)
Nakayama et al. [31]	Preservation group (remnant preserved)	50	14/50 (28%)	12	Semitendinosus autograft	Good: 92%
	Non‐preservation group (no remnant preservation)	75	22/75 (29%)	12	Semitendinosus autograft	Good: 59%
Sato et al. [38]	AA group (anatomical attachment)	34	34/34 (100%)	14 (11–24)	Hamstring autograft	AM bundle: Good 28/34 (82.4%); Fair 6/34 (17.6%) PL bundle: Good 25/34 (73.5%); Fair 8/34 (23.5%)
	NA group (non‐anatomical attachment)	33	33/33 (100%)	14 (11–24)	Hamstring autograft	AM bundle: Good 20/33 (60.6%); Fair 11/33 (33.3%) PL bundle: Good 17/33 (51.5%); Fair 14/33 (42.4%)
	NR group (no remnant)	22	22/22 (100%)	14 (11–24)	Hamstring autograft	AM bundle: Good 7/22 (31.8%); Fair 12/22 (54.5%) PL bundle: Good 6/22 (27.3%); Fair 9/22 (40.9%)
Lee et al. [26]	Group RC (remnant continuity)	50	31/50 (62.0%)	23.9	Hamstring autograft	Good 28/31 (90.3%), Fair 2/31 (6.5%)
	Group RD (remnant discontinuity)	44	23/44 (52.3%)	18.8	Hamstring autograft	Good 19/23 (82.6%), Fair 2/23 (8.7%)
Kondo et al. [24]	Group P (remnant‐preserving)	81	62/81 (76.5%)	14 (11–24)	Semitendinosus autograft + polyester tape augmentation (Leeds‐Keio Artificial Ligament)	Excellent 59/62 (95.2%), Good 3/62 (4.8%)
	Group R (remnant‐resecting)	98	46/98 (46.9%)	14 (11–24)	Semitendinosus autograft + polyester tape augmentation (Leeds‐Keio Artificial Ligament)	Excellent 37/46 (80.4%), Good 8/46 (17.4%)
Lu et al. [27]	Group EF (existing footprint) remnant preserving	72	28/36 (77.8%)	~24 months	Hamstring autograft	A 17/28 (60.7%), B 9/28 (32.1%)
	Group BL (bony landmarks) remnant resecting	36	31/36 (86.1%)	~24 months	Hamstring autograft	A 17/31 (54.8%), B 9/31 (29.0%)

*Note*: Summary of second‐look arthroscopic findings across included studies, including synovial coverage grade (Crain classification where reported), cyclops lesion incidence and other intra‐articular observations. Data stratified by remnant preservation strategy (RP‐ACLR vs. NP‐ACLR) where comparative data were available.

Abbreviations: ACLR, anterior cruciate ligament reconstruction; BPTB, bone–patellar tendon–bone; Crain I–IV, synovial coverage classification (I: PCL–tibial bridge; II: roof–tibial bridge; III: lateral wall–tibial bridge; IV: no substantial remnant); NP‐ACLR, non‐remnant‐preserving ACLR; PCL, posterior cruciate ligament; RP‐ACLR, remnant‐preserving ACLR; SB, single‐bundle; ST, semitendinosus.

#### Synovial coverage and graft ligamentization

**Figure 6:**
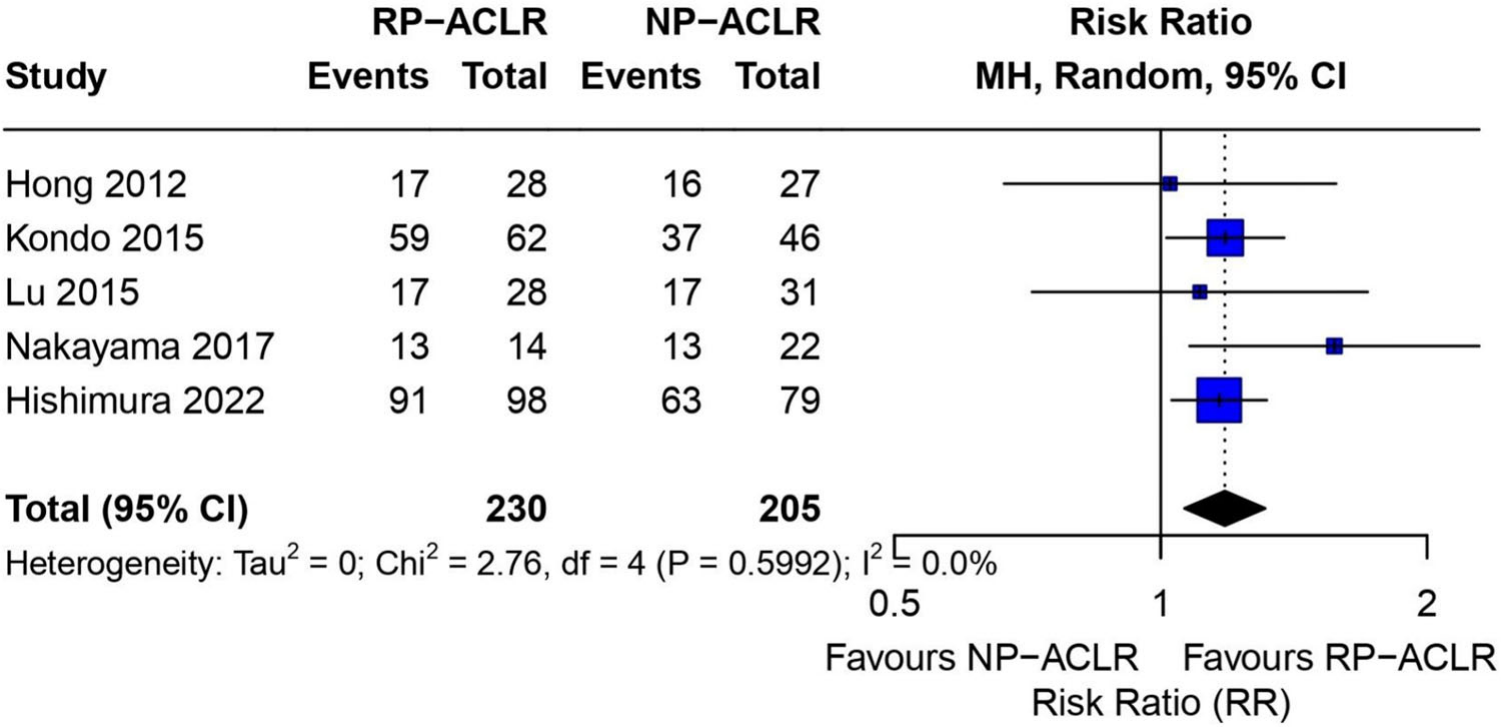
Synovial coverage: forest plot of RP‐ACLR versus NP‐ACLR. Forest plot comparing the probability of achieving the highest grade of synovial coverage at second‐look arthroscopy after remnant‐sparing (RP‐ACLR) versus non‐remnant‐sparing (NP‐ACLR) ACLR. Five studies were included (Hong 2012, Kondo 2015, Lu 2015, Nakayama 2017, Hishimura 2022; *n* = 230 RP‐ACLR knees vs. 205 NP‐ACLR knees). Study‐specific RRs and 95% CIs are shown as squares (size proportional to study weight) with horizontal lines; the combined estimate is shown as a diamond (Mantel–Haenszel, random‐effects model). A vertical line at RR = 1 indicates no between‐group difference; values > 1 favour RP‐ACLR. Inter‐study heterogeneity was low (*τ* ^2^ = 0; *χ* ^2 ^= 2.76, df = 4, *p* = 0.599; *I* ^2 ^= 0.0%). CI, confidence interval; df, degrees of freedom; *I* ^2^, heterogeneity statistic; NP‐ACLR, non‐remnant‐preserving ACLR; RP‐ACLR, remnant‐preserving ACLR; RR, risk ratio; *τ* ^2^, between‐study variance.

In combined analyses, RP‐ACLR showed a clear trend toward superior synovial coverage compared to techniques involving remnant resection or non‐anatomical remnant ligation. In all nine studies reporting measurable synovial coverage, the ‘good to excellent’ coverage rate (most commonly defined as graft coverage greater than 75%) was approximately 85% in the RP groups, while significantly lower and more variable rates (approximately 18.2%–79.7%) were observed in the comparison groups. Comparative studies consistently supported preservation: Nakayama et al. reported a 92.9% good coverage rate in the preservation group, compared to 59.1% in the NP group, and Hishimura et al. observed a 92.9% coverage rate in the preservation group (Crain I–III), compared to 79.7% in the resection group (Crain IV) [12, 31]. Forest plot comparisons of synovial coverage between RP‐ACLR and NP‐ACLR are presented in Figure 6.

**Figure 7:**
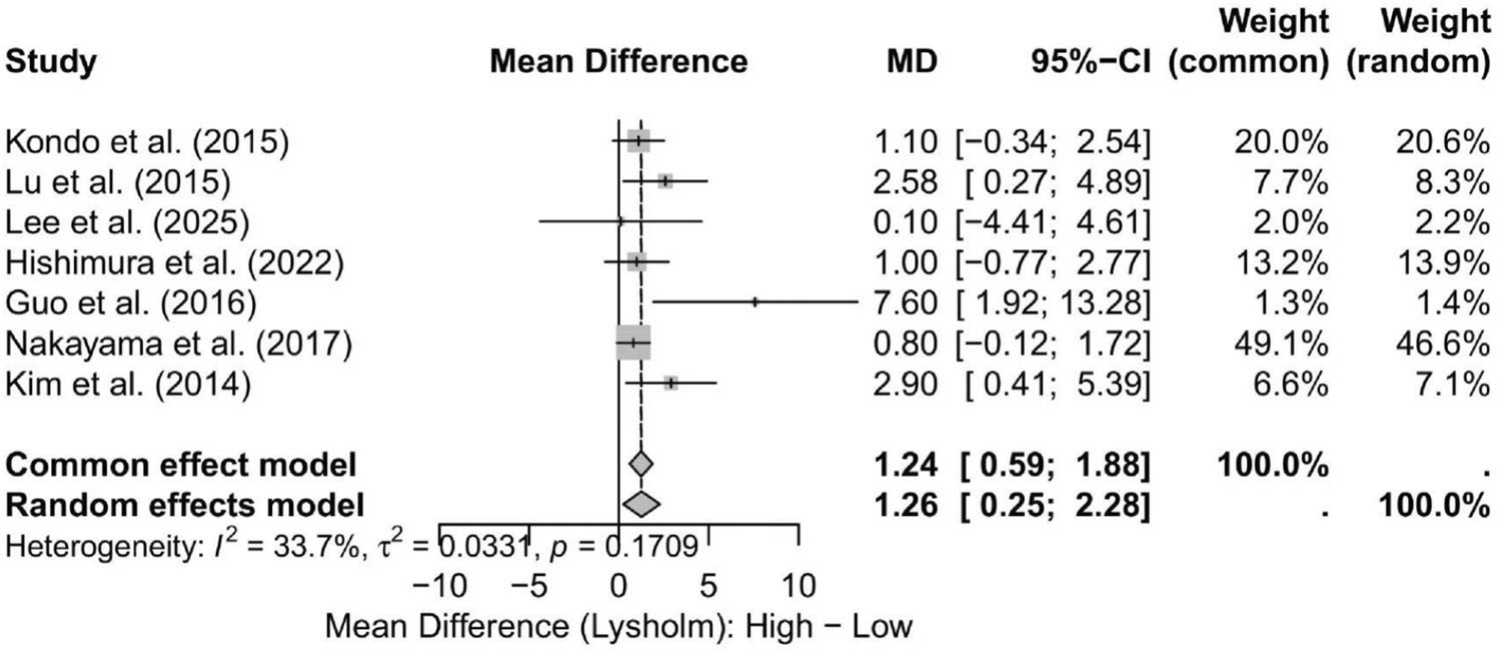
Lysholm scores stratified by synovial coverage grade. Forest plot of postoperative MDs in Lysholm scores between patients with higher versus lower synovial coverage at second‐look arthroscopy. Positive MD values indicate better Lysholm scores in the high‐coverage group. Square size is proportional to study weight; horizontal lines represent 95% CIs. Diamonds show pooled estimates: fixed‐effect MD = 1.24 (95% CI 0.59–1.88); random‐effects MD = 1.26 (95% CI 0.25–2.28). Between‐study heterogeneity was low to moderate (*I* ^2 ^= 33.7%, *τ* ^2 ^= 0.0331, *p* = 0.171). CI, confidence interval; *I* ^2^, heterogeneity statistic; MD, mean difference;*τ* ^2^, between‐study variance.

Furthermore, studies classifying the degree of preservation have suggested a dose–response relationship where higher synovial coverage rates are observed when a greater proportion of natural remnants are preserved. Noh et al. reported a good coverage rate of 90.7% when more than 75% of the remnant was preserved, and Kim et al. found a better coverage rate when more than 50% of the remnant was preserved (good coverage rate 54.5% and moderate/partial coverage rate 40.9%) compared to when 50% or less of the remnant was preserved [18, 33]. The relationship between synovial coverage and Lysholm functional outcomes is shown in Figure 7.

The pooled results are displayed as diamonds. Both models favoured the high‐coverage group: the fixed‐effect model showed an MD of 1.24 (95% CI 0.59–1.88), and the RE model showed an MD of 1.26 (95% CI 0.25–2.28). Between‐study heterogeneity was low to moderate (*I* ^2^ = 33.7%, *τ* ^2^ = 0.0331, *p* = 0.1709), suggesting limited inconsistency across studies.

**Figure 8:**
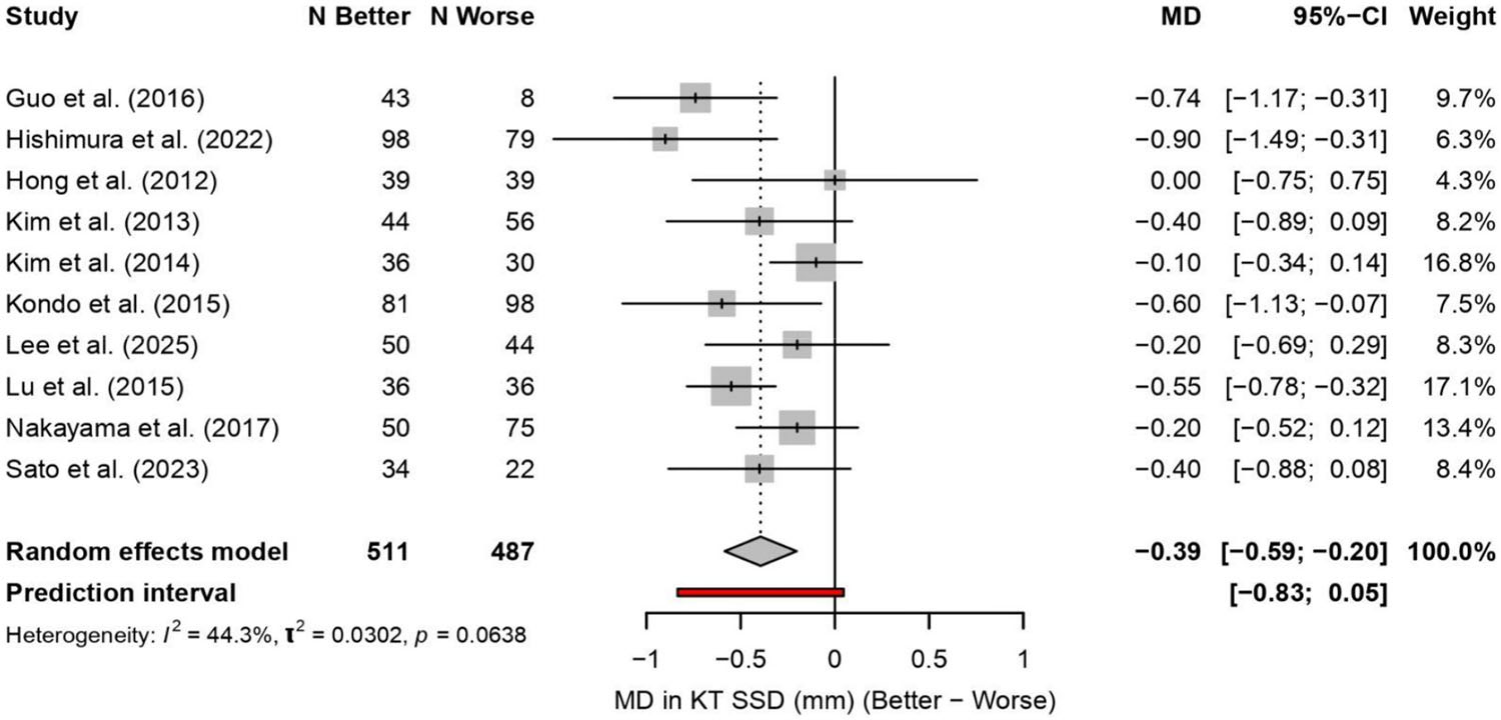
KT‐1000/2000 side‐to‐side difference stratified by synovial coverage grade. Forest plot of the association between synovial coverage grade and postoperative KT‐1000/2000 side‐to‐side difference after ACLR. Ten comparative study cohorts were pooled (better coverage: *n* = 511; worse coverage: *n* = 487) using a random‐effects model. Better synovial coverage was associated with a lower side‐to‐side difference (combined MD = −0.39 mm; 95% CI −0.59 to −0.20), indicating improved objective anterior knee stability. Inter‐study heterogeneity was moderate (*I* ^2^ = 44.3%, *τ* ^2 ^= 0.0302; *Q*‐test *p* = 0.064). ACL, anterior cruciate ligament; CI, confidence interval; MD, mean difference; *I* ^2^, heterogeneity statistic; *τ* ^2^, between‐study variance.

In the synovial coverage subgroup analysis, 10 comparative study cohorts (better synovial coverage: *n* = 511; worse synovial coverage: *n* = 487) were combined using a random effects model. Better synovial coverage was associated with a lower postoperative KT‐1000/2000 side‐to‐side difference, indicating improved objective stability; the combined MD was −0.39 mm (better vs. worse; 95% CI −0.59 to −0.20) (Figure 8). Inter‐study heterogeneity was moderate (*I* ^2^ = 44.3%, *τ* ^2^ = 0.0302; *Q*‐test *p* = 0.0638), indicating some variability in effect size between studies, while the overall trend consistently supported better synovial coverage.

#### Incidence of cyclops lesion

**Figure 9:**
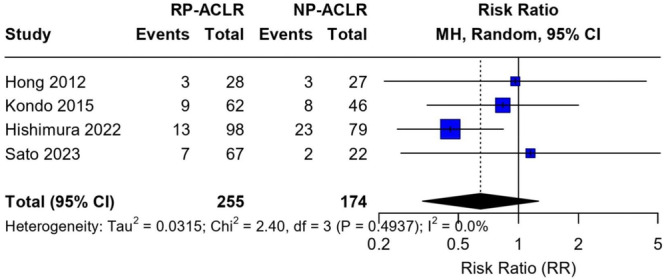
Cyclops lesion incidence: forest plot of RP‐ACLR versus NP‐ACLR. Forest plot comparing the risk of cyclops lesions on second‐look arthroscopy after remnant‐sparing (RP‐ACLR) versus non‐remnant‐sparing (NP‐ACLR) ACLR. Four studies were included (Hong 2012, Kondo 2015, Hishimura 2022, Sato 2023; *n* = 255 RP‐ACLR knees vs. 174 NP‐ACLR knees). Study‐specific RRs and 95% CIs are shown as squares with horizontal lines; the combined estimate (Mantel–Haenszel, random‐effects) is shown as a diamond. A vertical line at RR = 1 indicates no between‐group difference. The arrow indicates a CI extending beyond the plotted *x* axis range. Inter‐study heterogeneity was low (*τ* ^2 ^= 0.0315; *χ* ^2 ^= 2.40, df = 3, *p* = 0.494; *I* ^2 ^= 0.0%). ACLR, anterior cruciate ligament reconstruction; CI, confidence interval; df, degrees of freedom; *I* ^2^, heterogeneity statistic; NP‐ACLR, non‐remnant‐preserving ACLR; RP‐ACLR, remnant‐preserving ACLR; RR, risk ratio; *τ* ^2^, between‐study variance.

Across eight studies, cyclops lesions were identified at second‐look arthroscopy in approximately 15% of patients. Limited comparative data suggest lower cyclops rates with remnant preservation, as reported by Hishimura et al. (13.3% vs. 29.1% for preservation vs. resection), and potential variation according to remnant morphology or continuity (e.g., 10.0% with continuity vs. 6.8% with discontinuity in Lee et al.) [12, 26]. Overall, remnant characteristics and handling may influence cyclops formation, although these observations should be interpreted cautiously due to confounding and selection inherent in second‐look cohorts (Figure 9).

#### Overall complications

Across the included studies, reoperation for any reason represented the most frequent postoperative event, with a pooled incidence of 23.1% (95% CI, 9.6%–45.9%; *k* = 13), and substantial heterogeneity among studies (*I* ^2^ = 94.1%, *p* < 0.001). Cyclops lesion was the second most commonly reported complication, occurring in 9.4% of patients (95% CI, 6.1%–14.3%; *k* = 21), also with considerable between‐study heterogeneity (*I* ^2^ = 83.5%, *p* < 0.001). Other reported complications, including meniscal, chondral, and synovial pathologies, demonstrated a pooled rate of 7.0% (95% CI, 4.5%–10.7%; *k* = 9; *I* ^2^ = 68.0%, *p* = 0.0015).

Revision ACLR was required in 6.6% of cases (95% CI, 4.1%–10.4%; *k* = 9), with moderate heterogeneity (*I* ^2 ^= 61.9%, *p* = 0.007), while graft re‐rupture or failure occurred in 6.4% (95% CI, 5.4%–7.6%; *k* = 19) and showed no significant heterogeneity across studies (*I* ^2 ^= 0%, *p* = 0.54), indicating consistent reporting of this outcome (Table 5). Extension loss was observed in 6.4% of patients (95% CI, 3.7–10.9%; *k* = 5) with low‐to‐moderate heterogeneity (*I* ^2^ = 31.6%, *p* = 0.21).

**Table 5:** Meta‐analysis of complication rates after RP‐ACLR.

Outcome	Studies (*k*)	Pooled rate (%), random‐effects	95% CI	*I* ^2^ (%)	*Q p* Value
Reoperation (any reason)	13	23.1	9.6–45.9	94.1	<0.001
Overall complications	5	11.4	3.7–30.4	86.6	<0.001
Cyclops lesion	21	9.4	6.1–14.3	83.5	<0.001
Other complications^a^	9	7.0	4.5–10.7	68.0	0.0015
Revision ACLR	9	6.6	4.1–10.4	61.9	0.0072
Graft re‐rupture/failure	19	6.4	5.4–7.6	0.0	0.54
Extension loss	5	6.4	3.7–10.9	31.6	0.21

*Note*: Pooled incidence rates for postoperative complications following RP‐ACLR, estimated using RE meta‐analysis of proportions. Outcomes include reoperation (any reason), overall complications, cyclops lesion, other complications (meniscal re‐injury, chondral lesions, synovitis, hardware issues, donor‐site morbidity), revision ACLR, graft re‐rupture or failure, and extension loss. *I* ^2^ and *Q* test *p* values reflect between‐study heterogeneity. Other complications include meniscal re‐injury, chondral lesions, synovitis, hardware issues, and donor‐site morbidity.

Abbreviations: ACLR, anterior cruciate ligament reconstruction; CI, confidence interval; *I* ^2^, heterogeneity statistic; *k*, number of studies; RE, random‐effects; RP‐ACLR, remnant‐preserving ACLR.

^a^ Other complications include meniscal re‐injury, chondral lesions, synovitis, hardware issues and donor‐site morbidity.

When studies reporting composite outcomes were pooled, the overall complication rate was 11.4% (95% CI, 3.7–30.4%; *k* = 5), although this estimate was characterized by marked heterogeneity (*I* ^2^ = 86.6%, *p* < 0.001). Overall, the analysis demonstrates that reoperation and cyclops lesion are the most frequent adverse events following RP‐ACLR, with substantial interstudy variability, whereas graft failure rates appear comparatively consistent across the literature.

#### Allograft subgroup analysis

Four studies involving 535 patients evaluated remnant‐sparing ACLR using allograft tissue [11, 13, 18, 33]. The mean follow‐up period for these studies was 27 months, with an overall graft failure rate of 4.9% and a reoperation rate of 2.2%. Most secondary interventions were not related to graft failure; instead, they aimed to address complications in the meniscus or cartilage. Reported complications were rare (4%–5%) and mostly consisted of cyclops lesions or localized impingement. Importantly, no infection or graft rejection was reported in any of the included studies.

Hong et al. demonstrated that remnant‐sparing allograft reconstruction provided over 70% synovial coverage on second‐look arthroscopy [13], consistent with the pooled findings presented in Figure 6. This confirmed the biological benefit of preserving native tissue for revascularization and allograft integration.

#### RTS—Comparative outcomes

**Figure 10:**
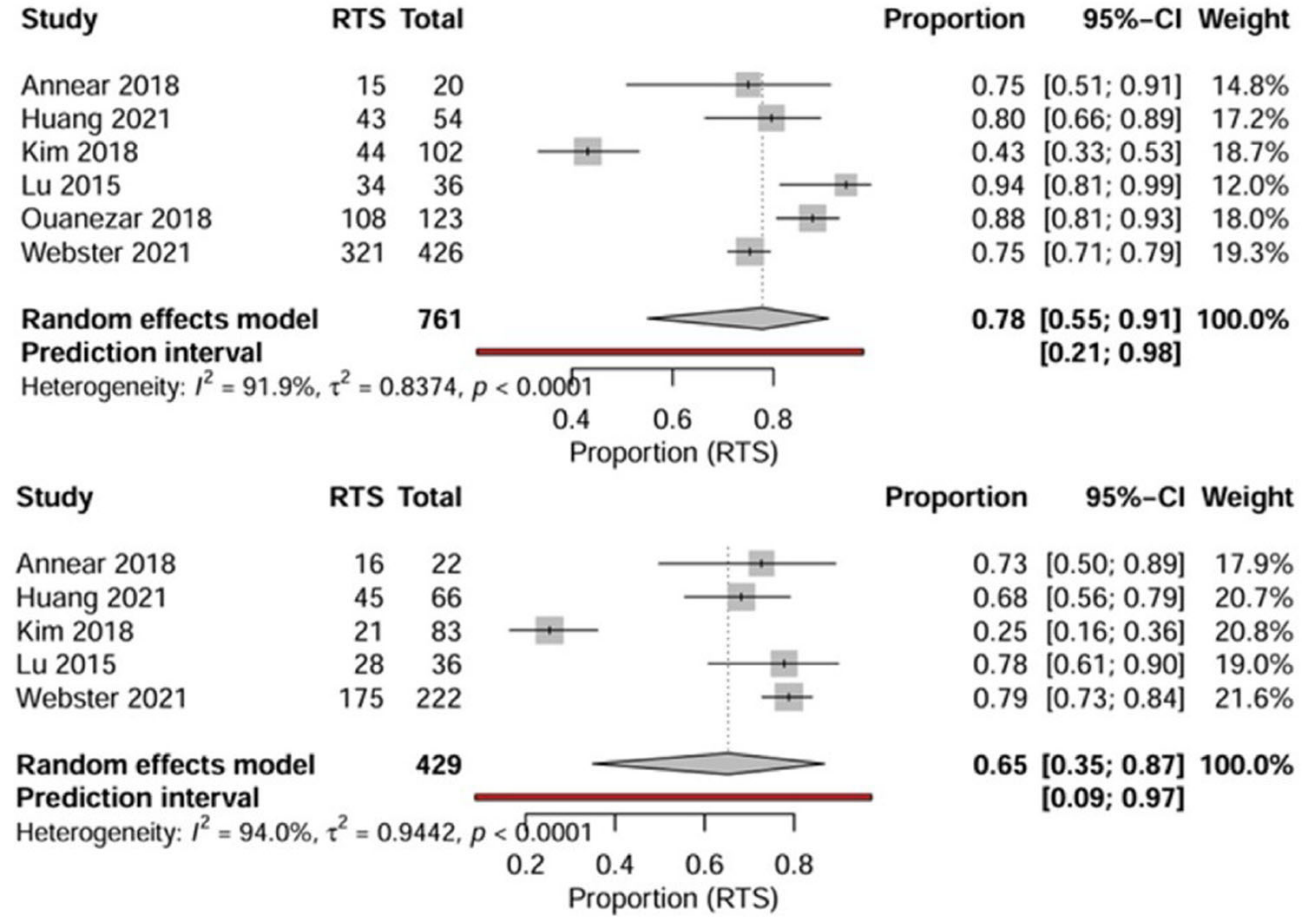
RTS rates: forest plots for RP‐ACLR and NP‐ACLR. Pooled RTS proportions after RP‐ACLR (upper panel) and NP‐ACLR (lower panel) ACLR. Random‐effects meta‐analysis of proportions with inverse‐variance weighting. Combined RTS rate: RP‐ACLR = 0.78 (95% CI 0.55–0.91; *I* ^2 ^= 91.9%); NP‐ACLR = 0.65 (95% CI 0.35–0.87; *I* ^2 ^= 94.0%). Substantial heterogeneity reflects variation in patient populations, RTS definitions, and follow‐up duration across included studies. ACLR, anterior cruciate ligament reconstruction; CI, confidence interval; *I* ^2^, heterogeneity statistic; NP‐ACLR, non‐remnant‐preserving ACLR; RP‐ACLR, remnant‐preserving ACLR; RTS, return to sport.

In the reviewed studies, RTS rates varied between 70% and 90%, with the majority of patients resuming prior activity levels within 6–9 months post‐surgery. Comparative data indicate that RP‐ACLR may enhance both the speed and level of RTS compared to NP‐ACLR approaches (Figure 10).

In a prospective cohort, Takazawa et al. demonstrated significantly higher RTS and activity levels in the RP group, with a mean Tegner score of 6.0 versus 5.1 in the NP‐ACLR group, alongside lower graft re‐rupture rates (1.1% vs. 7.1%) [43]. Similarly, Franciozi et al. found that patients with functionally preserved remnants achieved superior IKDC and Lysholm scores, reflecting better subjective knee function and readiness for sport [10]. Koga and Muneta and Huang et al. further supported this finding, showing faster recovery of knee stability and proprioception—both essential determinants for early and safe RTS [22].

## DISCUSSION

This systematic review and meta‐analysis demonstrates that RP‐ACLR may be associated with a significantly lower risk of graft re‐rupture compared with standard remnant‐sacrificing techniques. Although the biological rationale for remnant preservation is well established, previous syntheses have been limited by methodological heterogeneity and inconsistent reporting of safety outcomes, leaving the comparative risks of graft failure and reoperation incompletely defined. The present study, representing the largest pooled analysis to date focused on complications, provides robust evidence that RP‐ACLR not only maintains a comparable safety profile but also confers a clinically meaningful protective effect against graft failure.

The studies included in this systematic review demonstrate that RP‐ACLR maintains a complication profile comparable to, and in many respects superior to, standard remnant‐sacrificing procedures. Overall complication rates across studies typically range from 0% to 15%, with no evidence of a systematic increase attributable to remnant preservation itself [9]. More commonly reported minor adverse events included transient stiffness and asymptomatic cyclops lesions, while serious events like deep infection remain rare (0.5%–2%). This is in keeping with previously reported systematic reviews [36].

### Reoperation rate

The present systematic review demonstrates that reoperation rates for RP‐ACLR are generally favourable. When studies involving mandatory second‐look surgeries or routine equipment removal are excluded from the protocol [13, 18], the mean reoperation rate drops to a clinically acceptable 4%–7%. This rate is comparable to, and even lower than, standard ACLR rates in some key studies. Van Keulen et al. reported a 2‐year revision ACLR rate of only 2% in the remnant stretching group, significantly lower than the 18% in the standard resection group [17]. This was corroborated by Takazawa et al., who found graft rupture rates of 1.1% in the RP group and 7.1% in the non‐RP group [43]. This evidence strongly suggests that the primary long‐term benefit of RP‐ACLR may be a significant reduction in the need for revision surgery due to graft failure.

### Cyclops lesions

The theoretical risk of arthrofibrosis and cyclops lesion formation in ACLR is central to the debate. A complex picture emerges from this review. Some included studies, such as Nakayama et al., have found a higher rate of extension loss requiring debridement (12%) in RP groups, but this has not been found in other studies [31]. Conversely, Webster et al. found a negative correlation and determined that the likelihood of needing surgery for symptomatic cyclops lesions was lower when more than 50% of the tibial stump was preserved (1.1%) than when the stump was not preserved (5.3%) [44]. In the allograft‐based series examined in this review, the incidence of symptomatic cyclops lesions was consistently low, ranging from 1.5% to 0% [19, 33]. All cases were also effectively managed with mild arthroscopic excision, leading to minimal remnant stiffness. This suggests that the risk is related more to the surgical technique than to the preservation procedure itself, particularly to the proper shaping or cutting of the remaining portion to avoid stretching and notch impingement. Therefore, the risk of a clinically significant cyclops lesion requiring intervention appears low and manageable with appropriate treatment.

### Allograft outcomes and comparison with autograft reconstructions

A key aim of this review was to ascertain whether allografts improve graft survival when used in RP ACL surgery. The pooled analysis of four clinical studies encompassing a total of 535 patients demonstrates that RP‐ACLR using allografts yields consistently favourable short‐to mid‐term outcomes. With an average follow‐up period of approximately 32 months, the overall graft failure rate was 4.9% and the reoperation rate was 2.9%, primarily due to secondary procedures addressing meniscal or chondral pathology rather than graft‐related issues. Complications were infrequent, occurring in approximately 4%–5% of cases, and typically consisted of cyclops lesions or impingement. Importantly, no study reported infection, graft rejection, or other serious adverse events, underscoring the biological safety of the allograft approach.

Pooled analysis indicates that the rates of revision and re‐rupture in patients receiving allografts were similar to those in autograft recipients at equivalent mean follow‐up periods. In particular, the allograft studies reported re‐rupture rates of 5.3% and 7.8%, respectively [11, 19] these numbers fall within the broad range of failure rates reported in autograft studies (e.g., 1.1% in Takazawa 2013 to 13.6% in Annear 2018) [3, 43]. These results indicate that, in the short to medium term, graft survival rates are similar across allograft and autograft reconstructions. The failure rates of allografts in ACLR vary by graft type, patient‐related factors, timing and level of resumed activity. Pooled failure rates for soft tissue‐only allografts in adults can reach 20% and 11% for bone‐soft tissue allografts in adults, according to meta‐analyses [42] higher rates are observed in younger, more active people [8]. Long‐term studies demonstrate that allograft failure can reach 25% in young athletic populations, exceeding three times the failure rate of autografts [5]. Revision rates for allografts are consistently higher than for autografts, especially in patients with high demand, and other factors like donor characteristics and graft processing methods also affect results [8, 28].

The present meta‐analyses also revisit the debate on graft selection in ACLR. While graft material selection appears to have no statistically significant effect on re‐rupture rates, the remnant preservation surgical technique emerged as a critical factor associated with a threefold reduction in failure risk in hamstring autografts (approximately 2.8% vs. approximately 8.9%). This suggests that optimizing the biological environment through remnant preservation may outweigh the importance of graft type alone. Future surgical strategies should prioritize standardized remnant preservation techniques alongside graft selection considerations.

To date, no studies have reported long‐term outcomes of RP‐ACLRs utilizing allografts. Consequently, it is yet too early to make statistical inferences on how remnant preservation can improve allograft survival. To minimize donor‐site morbidity and maintain isokinetic balance, allografts can be still considered as a viable treatment option. Prolonged allograft survival may be achievable through RP techniques combined with additional orthobiologic treatments. Further research and advancement of technology are required to validate and optimize this approach.

A key biological advantage of the RP technique is its promotion of synovial coverage and revascularization of the graft. Studies employing second‐look arthroscopy, such as that by Hong et al., demonstrated up to 71% synovial coverage following allograft reconstruction—an indication of successful surface healing and ligamentization [13]. Similarly, Guo et al. observed that patients with well‐preserved remnants showed superior synovialization and knee stability compared with those with poor remnant quality, in whom early graft failure was more frequent [11]. Beyond the direct RP versus NP comparison, a multilevel synthesis of postoperative IKDC scores according to top‐grade synovial coverage (pooled mean: 79.07, 95% CI 73.21–84.92) suggested that higher synovial coverage grades tend to be associated with better patient‐reported function, although considerable variability across study groups precluded definitive conclusions. These findings suggest that remnant preservation supports a biologically active environment that facilitates graft maturation and integration, effectively compensating for the slower intrinsic healing capacity of allograft tissue. When compared to autograft RP techniques, allograft reconstructions demonstrate comparable short‐ to mid‐term clinical outcomes, particularly in terms of knee stability, patient‐reported function and complication rates. Although autografts are traditionally favoured for their superior long‐term survivorship, remnant preservation appears to substantially narrow the performance gap by enhancing the biological incorporation of allograft tissue. The improved synovial coverage observed in allograft cases may play a pivotal role in this process, promoting earlier revascularization and reducing tunnel widening—features that are critical for long‐term graft integrity. Nonetheless, long‐term data comparing the two graft types within RP frameworks remain limited. While autografts continue to demonstrate lower re‐rupture rates beyond 5 years in standard reconstructions, no studies have yet reported long‐term (>5‐year) outcomes of RP allograft ACLR. Thus, although the current evidence strongly supports the short‐ to mid‐term efficacy and safety of RP allograft ACLR, further longitudinal research is needed to determine whether its biological advantages—particularly enhanced synovialization—translate into sustained mechanical durability and graft survival over time.

### Strengths and limitations

This systematic review and meta‐analysis has several notable strengths. It represents the largest pooled quantitative synthesis to date specifically focused on complications, reoperation and graft failure after RP‐ACLR, incorporating robust RE modelling, Hartung–Knapp adjustment and CRVE‐based meta‐regression to account for clustering and small‐study effects. Comprehensive subgroup and moderator analyses (graft type, bundle configuration, remnant morphology, synovial coverage, BMI and surgical timing) allowed an in‐depth exploration of potential sources of heterogeneity, while strict PRISMA adherence and formal ROBINS‐I/RoB 2 assessments enhanced methodological rigour.

Several limitations should also be acknowledged. Most included studies were non‐randomized, with moderate risk of confounding and selection bias and surgical techniques, remnant classification systems and outcome definitions were heterogeneous. Second‐look arthroscopy data were available only in selected cohorts, introducing potential selection bias. The number of studies available for certain meta‐regression analyses (e.g., BMI, surgical delay) was limited, restricting statistical power to detect small or nonlinear effects. Finally, the evidence bases predominantly represented young, normal‐weight, physically active populations, limiting generalizability to older, obese or low‐demand patients. These factors should be considered when interpreting the magnitude and external validity of the observed protective effect of remnant preservation.

## CONCLUSION AND CLINICAL IMPACT

In conclusion, this systematic review and meta‐analysis confirm RP‐ACLR as a safe and clinically advantageous evolution in surgical technique. The synthesis demonstrates that RP‐ACLR significantly reduces the risk of graft re‐rupture and does not increase the overall burden of complications or reoperations compared to standard techniques. While vigilant surgical technique to fashion the remnant and avoid impingement remains important, these are modifiable considerations.

The risk‐benefit profile strongly favours remnant preservation in appropriately selected patients. The technique exchanges a minimal and manageable potential for minor complications for a substantial reduction in the most serious adverse event—graft failure and subsequent complex revision surgery. For the practicing surgeon, incorporating RP‐ACLR where feasible offers a viable pathway to enhance graft survival, improve structural outcomes and support a higher level of functional recovery. Future research should focus on standardizing indications based on remnant quality and elucidating the precise technical nuances that optimize long‐term results.

## AUTHOR CONTRIBUTIONS

**Ozgur Basal**: Conceptualization; methodology; investigation; formal analysis; writing—original draft preparation; writing—review and editing. **James G. Jefferies**: Methodology; data curation; writing—original draft preparation. **Jure Serdar**: Investigation; data curation; validation; visualization; writing—review and editing. **Emmanuel Papakostas**: Investigation; validation; resources; writing—review and editing. **Furkan Karakas**: Formal analysis; data curation; visualization; writing—review and editing. **Gazi Huri**: Supervision; project administration; methodology; writing—review and editing. **Mahmut Nedim Doral**: Conceptualization; supervision; funding acquisition; resources; writing—review and editing; final approval. All authors have read and agreed to the final version of the manuscript.

## CONFLICT OF INTEREST STATEMENT

The authors declare no conflicts of interest.

## ETHICS STATEMENT

Ethical approval was not required for this systematic review and meta‐analysis.

## Data Availability

Data extracted from published studies are available from the corresponding author upon reasonable request.
